# Computational Repurposing of Drugs and Natural Products Against SARS-CoV-2 Main Protease (M^pro^) as Potential COVID-19 Therapies

**DOI:** 10.3389/fmolb.2022.781039

**Published:** 2022-03-14

**Authors:** Sakshi Piplani, Puneet Singh, Nikolai Petrovsky, David A. Winkler

**Affiliations:** ^1^ College of Medicine and Public Health, Flinders University, Bedford, SA, Australia; ^2^ Vaxine Pty Ltd., Warradale, SA, Australia; ^3^ Department of Biochemistry and Chemistry, La Trobe Institute for Molecular Science, La Trobe University, Melbourne, VIC, Australia; ^4^ Monash Institute of Pharmaceutical Sciences, Monash University, Parkville, VIC, Australia; ^5^ School of Pharmacy, University of Nottingham, Nottingham, United Kingdom

**Keywords:** SARS-CoV-2, binding affinity, main protease, 3CL, computational chemistry, docking, molecular dynamics

## Abstract

We urgently need to identify drugs to treat patients suffering from COVID-19 infection. Drugs rarely act at single molecular targets. Off-target effects are responsible for undesirable side effects and beneficial synergy between targets for specific illnesses. They have provided blockbuster drugs, e.g., Viagra for erectile dysfunction and Minoxidil for male pattern baldness. Existing drugs, those in clinical trials, and approved natural products constitute a rich resource of therapeutic agents that can be quickly repurposed, as they have already been assessed for safety in man. A key question is how to screen such compounds rapidly and efficiently for activity against new pandemic pathogens such as SARS-CoV-2. Here, we show how a fast and robust computational process can be used to screen large libraries of drugs and natural compounds to identify those that may inhibit the main protease of SARS-CoV-2. We show that the shortlist of 84 candidates with the strongest predicted binding affinities is highly enriched (≥25%) in compounds *experimentally* validated *in vivo* or *in vitro* to have activity in SARS-CoV-2. The top candidates also include drugs and natural products not previously identified as having COVID-19 activity, thereby providing leads for experimental validation. This predictive *in silico* screening pipeline will be valuable for repurposing existing drugs and discovering new drug candidates against other medically important pathogens relevant to future pandemics.

## Introduction

The devastating impact of the COVID-19 pandemic caused by SARS coronavirus-2 (SARS-CoV-2) has stimulated unprecedented international activity to discover effective drugs for this and other pathogenic coronaviruses such as SARS and MERS CoV (Ciotti et al., 2019; Zhang J. et al., 2020; Berkley, 2020; Zhang J.-J. et al., 2020; Zhang T. et al., 2020; Jiang, 2020; Lu, 2020; Mendes, 2020; Olsen et al., 2020; Rosa and Santos, 2020; Rosales-Mendoza et al., 2020; Sanders et al., 2020; Schlagenhauf et al., 2020; Sohrabi et al., 2020; Thanh Le et al., 2020; Whitworth, 2020; Yavuz and Unal, 2020). Computational methods are useful, fast approaches to determine the affinities of small drug-like molecules for SARS-CoV-2 protein targets. Recent papers in Science have reported effective computational *de novo* drug design based on the structures of the SARS-CoV-2 protease (Zhang L. et al., 2020; Dai et al., 2020). Clearly, the design of potent new drugs for coronaviruses is very important for future pandemic preparedness, given that the last three serious epidemics have been caused by coronaviruses. However, to make an impact on the current COVID-19 pandemic, given the 10- to 15-year time frame required to take drug leads from lab to clinic, it is only feasible to repurpose drugs that are already registered (off label use), have been through at least phase 1 clinical trials to establish initial human safety, or are approved natural products. Any COVID-19 drug candidates identified in this way can then be used very quickly, as their safety and pharmacokinetics should be already well understood. Drugs that reduce viral replication primarily by targeting viral proteases and polymerases are classified as direct-acting antivirals and are the focus of the current work. Other studies have explored host-targeted drugs that inhibit cellular functions required for viral replication and thereby inhibit SARS-CoV-2 infection, albeit with more potential for host side effects(Saul and Einav, 2020).

**FIGURE 1:**
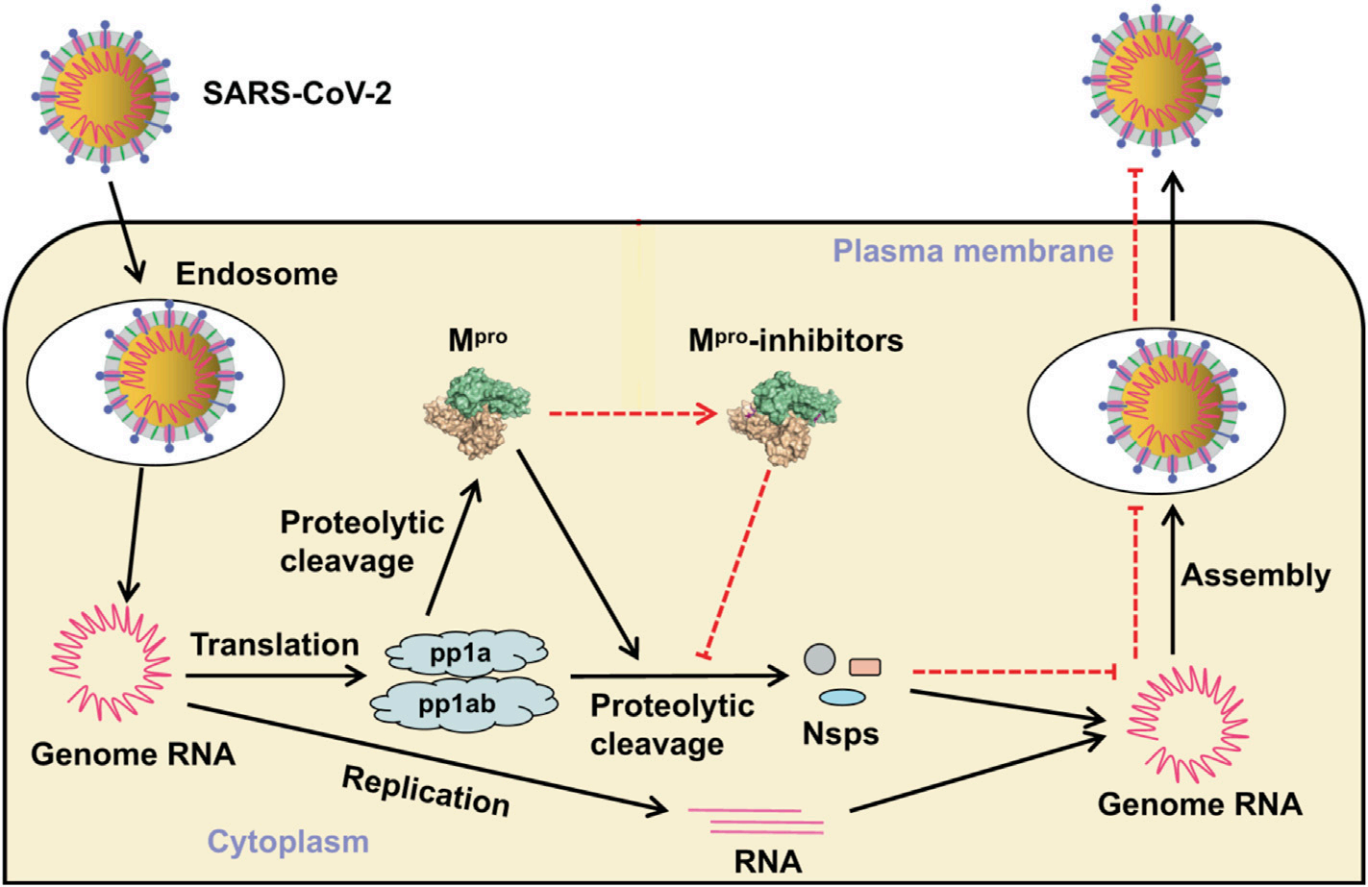
Virus entry and replicative cycle. M^pro^ produces non-structural proteins (Nsps) that are essential for assembly of the viral replication transcription complex needed for RNA synthesis. Inhibitors bind to M^pro^, resulting in failure of virion assembly and inhibited release of new virions. Adapted from Mengist et al. (2020) (https://creativecommons.org/licenses/by/4.0/).

The SARS-CoV-2 genome encodes >20 proteins, many of which are potential antiviral drug targets (Figure 1). Two proteases (PLpro and 3CLpro) are essential for virus replication. These enzymes cleave the PP1A and PP1AB polyproteins into functional components. 3-Chymotrypsin-like protease (3CLpro) catalytically self-cleaves a peptide bond between a glutamine at position P1 and a small amino acid (serine, alanine, or glycine) at position P1’. This protease corresponds to non-structural protein 5 (nsp5), the main protease (aka main protease, M^pro^) in coronaviruses. 3CL protease is crucial to the processing of the coronavirus replicase polyprotein (P0C6U8), cleaving it at 11 conserved sites. It employs a Cys-His catalytic dyad in its active site, where the cysteine sulfur is the nucleophile, and the histidine imidazole ring acts as a general base. M^pro^ is a conserved drug target present in all *Coronavirinae*. It does not have a human homolog, reducing the risk that drugs inhibiting it will exhibit side effects (Sheik Amamuddy et al., 2020). Very recent research has shown that strong M^pro^ inhibitors can substantially reduce SARS-CoV-2 virus titers, reduce weight loss, and improve survival in mice (Rathnayake et al., 2020), making M^pro^ a promising drug target for structure-based drug discovery.

Computational methods can rapidly and efficiently identify candidate drugs for repurposing in pandemic situations where speed is of utmost importance. A very recent paper by Llanos et al. analyzed the strengths and weaknesses of docking simulations for SARS-CoV-2 drug repurposing for M^pro^ (Llanos et al., 2021). This study disclosed that most published studies do not check the ability of the docking method to accurately redock ligands from protein structures and do not account for protein and ligand flexibility using MD calculations, and only a tiny percentage validate predictions using experimental measurements of virus activity. To address these shortcomings, here we used validated molecular docking followed by high-throughput molecular dynamics simulations to prioritize, from an initial large number of licensed or clinical trial drugs and natural products, a short list of the most promising candidates.

## Results

Molecular dynamics calculations were used to predict the optimal binding poses and binding energies for 84 of the top hits from docking-based virtual screening of ∼12,000 drug candidates against the SARS-CoV-2 M^pro^. The docking protocols were validated by redocking ligands from 10 x-ray structures. The top candidates were ranked for COVID-19 repurposing based on binding affinity and novelty. Conspicuously, we found that ∼30% of the computationally repurposed drug candidates have experimentally validated activity against the M^pro^ target protein, the SARS-CoV-2 virus, or both. Several of the drugs we identified are currently in clinical trials for COVID-19.

The binding energies of the 84 top ranked ligands from the docking calculations are listed in Supplementary Table S1. Note that calculating accurate *absolute* binding energies is difficult, and the approach we have taken provides good estimates of the *relative* binding energies of repurposing candidates. The ten drugs with the tightest binding to M^pro^ are summarized in Table 1, together with their GMXPBSA binding energies. The binding energies of several of the antiviral drugs, namely, simeprevir, sofosbuvir, lopinavir, and ritonavir, are very similar, within the uncertainties in calculated energies. Some of the antivirals were also identified in other *in silico* docking studies or wet-lab SARS-CoV-2 activity studies, as we discuss below. This, together with a subsequent extensive search of the literature for experimental data, provides strong validation of the utility of our computational methods to find leads consistent with other studies that also inhibit SARS-CoV-2 or the relevant protein target. It strongly suggests that the computational protocols we have adopted are very capable of generating a list of repurposing candidates, many of which are likely to exhibit useful experimental *in vitro* activity at least.

**TABLE 1:** Binding energies of 10 top ranked small-molecule ligands for SARS-CoV-2 M^pro^.

ID	Structure	Description	ΔG_MMPBSA_ (ΔG_bind_) (kcal/mol)
C3809489 bemcentinib	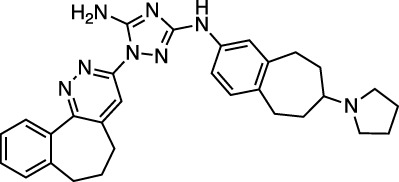	Inhibitor of the kinase domain of AXL receptor	−34.7 ± 2.6 (−30.7)
C4291143 PC786	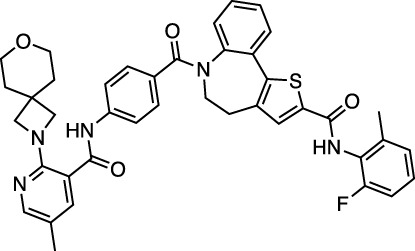	Respiratory syncytial virus (RSV) L protein polymerase inhibitor	−33.1 ± 0.3 (−29.2)
C787 Montelukast	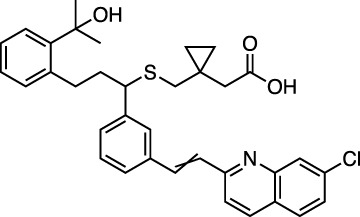	Leukotriene receptor antagonist used with cortico-steroids for asthma therapy	−32.7 ± 0.2 (−20.6)
C442 Ergotamine	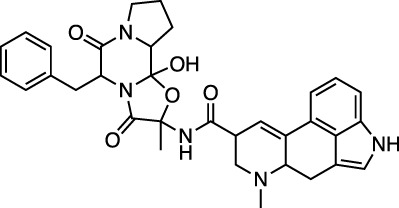	Alpha-1 selective adrenergic agonist used in migraine treatment	−31.5 ± 0.3 (−28.7)
D06290 simeprevir	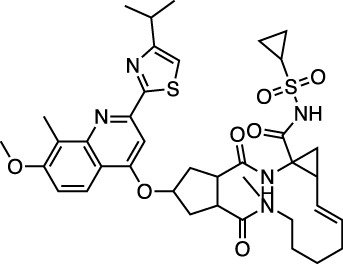	Hepatitis C virus (HCV) NS3/4A protease inhibitor	−31.4 ± 0.2 (−29.2)
D08934 sofosbuvir	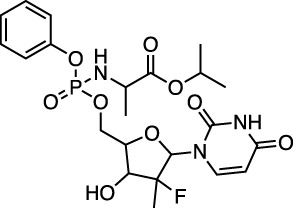	Nucleotide prodrug and HCV NS5B polymerase inhibitor	−31.0 ± 0.5 (−22.8)
D01601 lopinavir	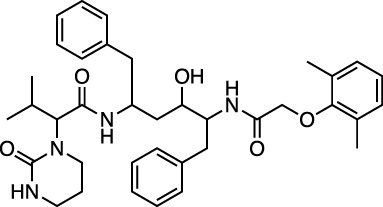	Antiretroviral protease inhibitor for treatment of HIV-1	−30.7 ± 0.3 (−20.4)
D00503 ritonavir	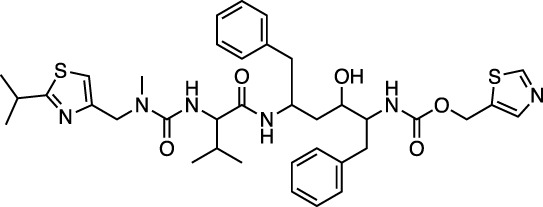	Peptidomimetic inhibitor of HIV-1 and HIV-2 proteases	−30.5 ± 0.5 (−21.3)
C2105887 Mergocriptine	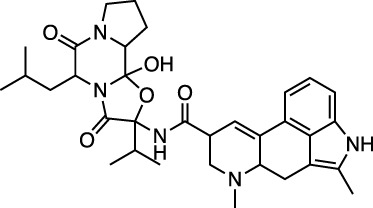	Synthetic ergot derivative, dopamine receptor agonist	−30.0 ± 0.3 (−17.9)
D14761 remdesivir	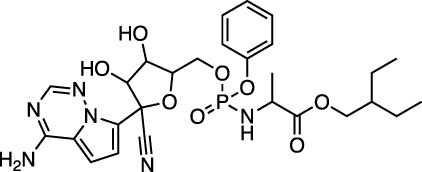	Viral RNA-dependent RNA polymerase inhibitor	−30.0 ± 0.2 (−27.1)

In this paper, we have focused particularly on tightly binding drugs with novel structures, such as ergot compounds, bemcentinib, PC786, and montelukast.

Although the main focus of the paper is to show that appropriate computational methods can make useful predictions of the repurposing potential of drugs, we also provide a preliminary analysis of the binding of candidate drugs to the M^pro^ active site. M^pro^ achieves protein cleavage *via* the catalytic dyad His41and Cys145. The main active site residues that have previously been implicated in drug binding are His41, Gly143, Cys145, His163, Glu166, and Glu166. All of the drugs whose interactions with the M^pro^ binding site are summarized below interact with these six residues (see Supplementary Table S2). Most form strong hydrogen binds to one or more of Gly143, Cys145, and His163. All docked and MD simulated structures of M^pro^ with the repurposed drug candidates were also deposited in open access data archives. Supplementary Figure S2 shows a superimposition of the top 10 drugs bound to the M^pro^ site.

**FIGURE 2:**
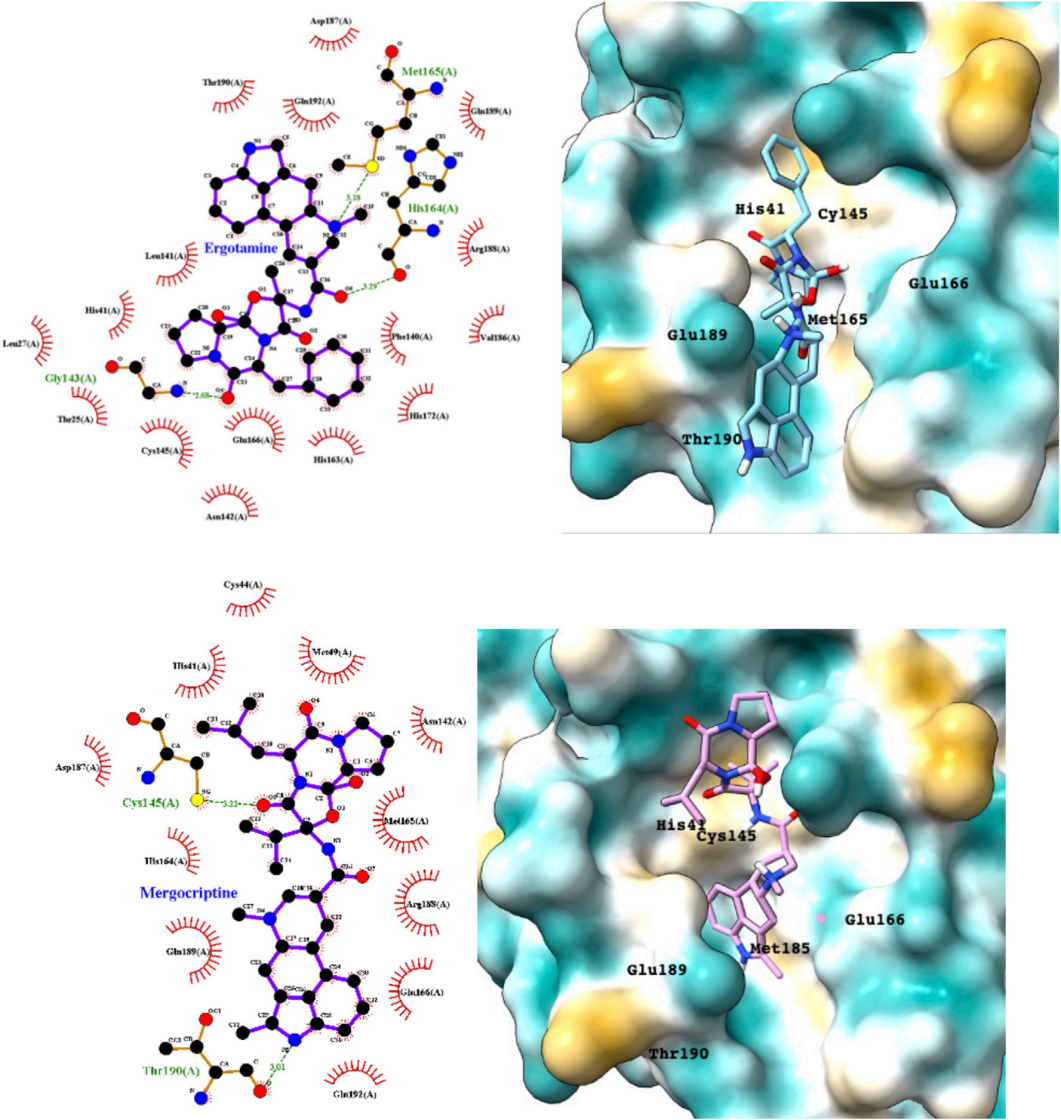
LigPlot (left) and hydrophobic protein surface representation (right) of the main interactions between M^pro^ and ergotamine (top) and mergocriptine (bottom). The molecular surface denotes hydrophobicity of the pockets (blue hydrophilic, yellow/brown hydrophobic). Key binding site residues are labeled to help orient the viewer.

Ergotamine and mergocriptine, a synthetic long-acting ergot derivative, are α1 selective adrenergic agonist vasoconstrictors and an agonist of dopamine receptors, respectively. Figure 2 shows a LigPlot representation of the interactions of key functional groups in ergotamine and mergocriptine with protease active site residues. These, together with the accompanying M^pro^ binding site molecular surface plots encoded for lipophilicity, illustrate how these drugs bind in the protease binding site. The specific interactions between these drugs and the residues in the binding site are summarized in Supplementary Table S2. Both drugs make strong and multiple interactions with 20 active site residues, notably hydrogen bonds with Gly143, His164, Met165, Cys145, and Thr190.

**FIGURE 3:**
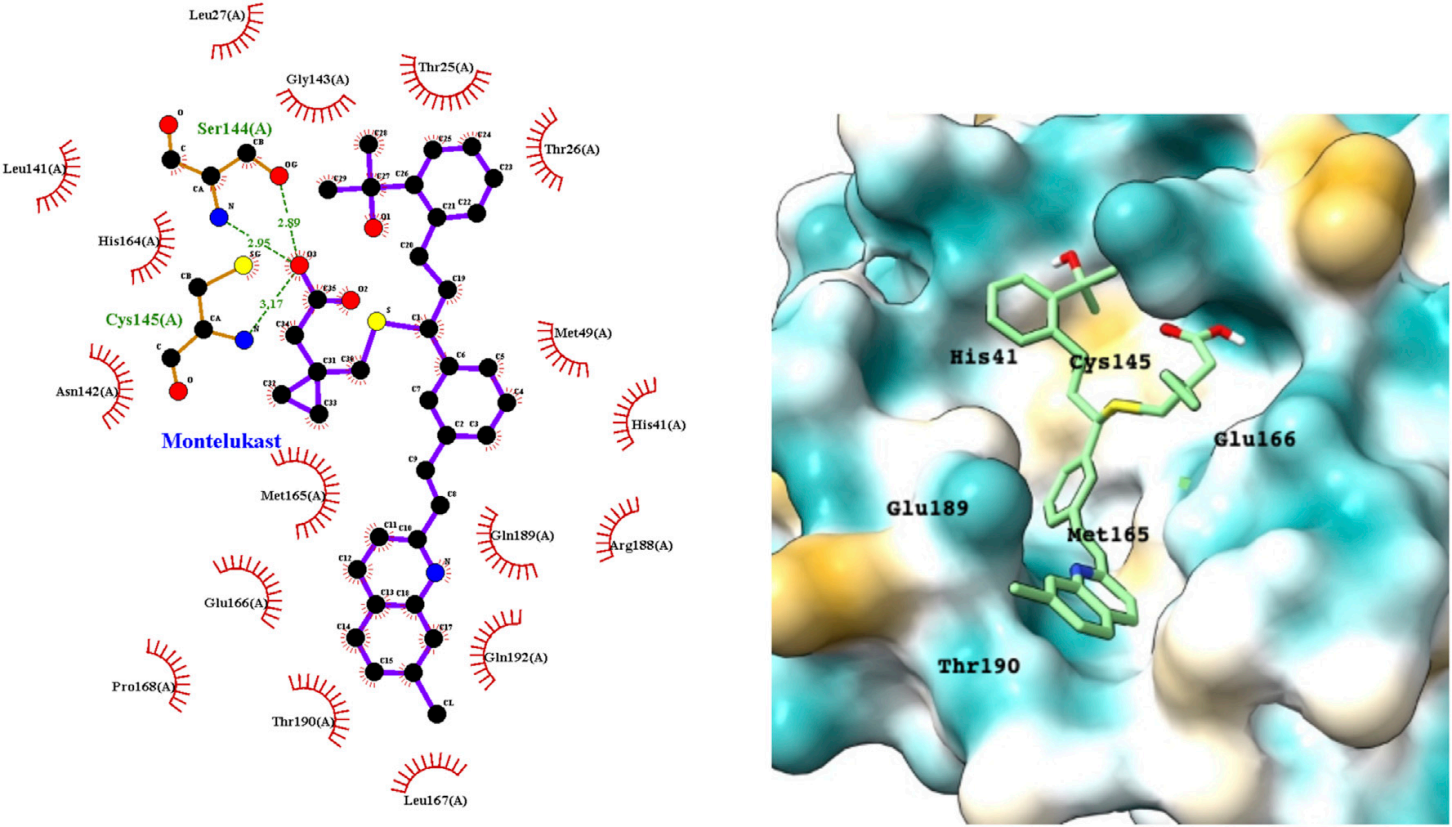
LigPlot (left) and hydrophobic protein surface representation (right) of the main interactions between M^pro^ and montelukast. The molecular surface denotes hydrophobicity of the pockets (blue hydrophilic, yellow/brown hydrophobic). Key binding site residues are labeled to help orient the viewer.

Montelukast is a cysteinyl leukotriene receptor antagonist used to treat asthma and allergic rhinitis. It reduces pulmonary responses to antigen, tissue eosinophilia and IL-5 expression in inflammatory cells and decreases elevated levels of IL-1β and IL8 in viral upper respiratory tract infections (Almerie and Kerrigan, 2020). Figure 3 shows a LigPlot representation of the interactions of key functional groups in montelukast with protease active site residues and a representation of how this drug binds in the active site of M^pro^. The specific interactions between montelukast and the active site residues are also summarized in Supplementary Table S2. The drug interacts extensively with the active site, binding to 23 residues, forming strong hydrogen bonds with Ser144 and Cys145. Montelukast spans the relatively broad binding pocket of the enzyme.

**FIGURE 4:**
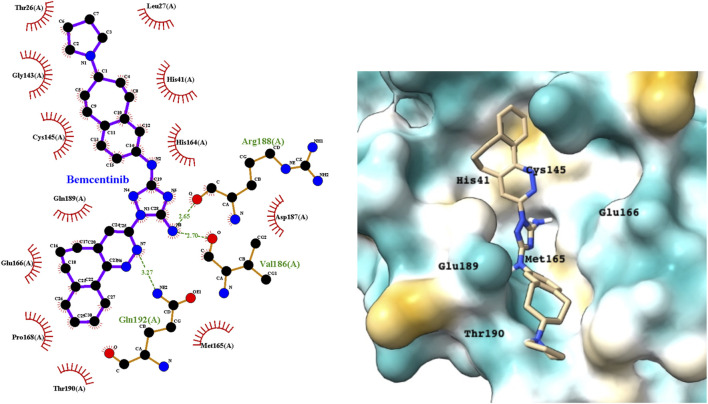
LigPlot (left) and hydrophobic protein surface representation (right) of the main interactions between M^pro^ and bemcentinib. The molecular surface denotes hydrophobicity of the pockets (blue hydrophilic, yellow/brown hydrophobic). Key binding site residues are labeled to help orient the viewer.

Bemcentinib selectively inhibits AXL kinase activity, which blocks viral entry and enhances the antiviral type I interferon response. Figure 4 provides a LigPlot representation of the interactions of key functional groups in bemcentinib with protease active site residues, which are also summarized in detail in Supplementary Table S2. It forms strong hydrogen-bonding interactions with Val186, Arg188, andGln192. Figure 4 also shows the docking pose of bemcentinib in the protease active site after simulation by MD. The hydrophobic benzocycloheptapyridazine moiety occupies a relative hydrophobic pocket, while the hydrophilic triazolyldiamine moiety binds strongly to the polar pocket formed partially by Asp187 and Arg188.

**FIGURE 5:**
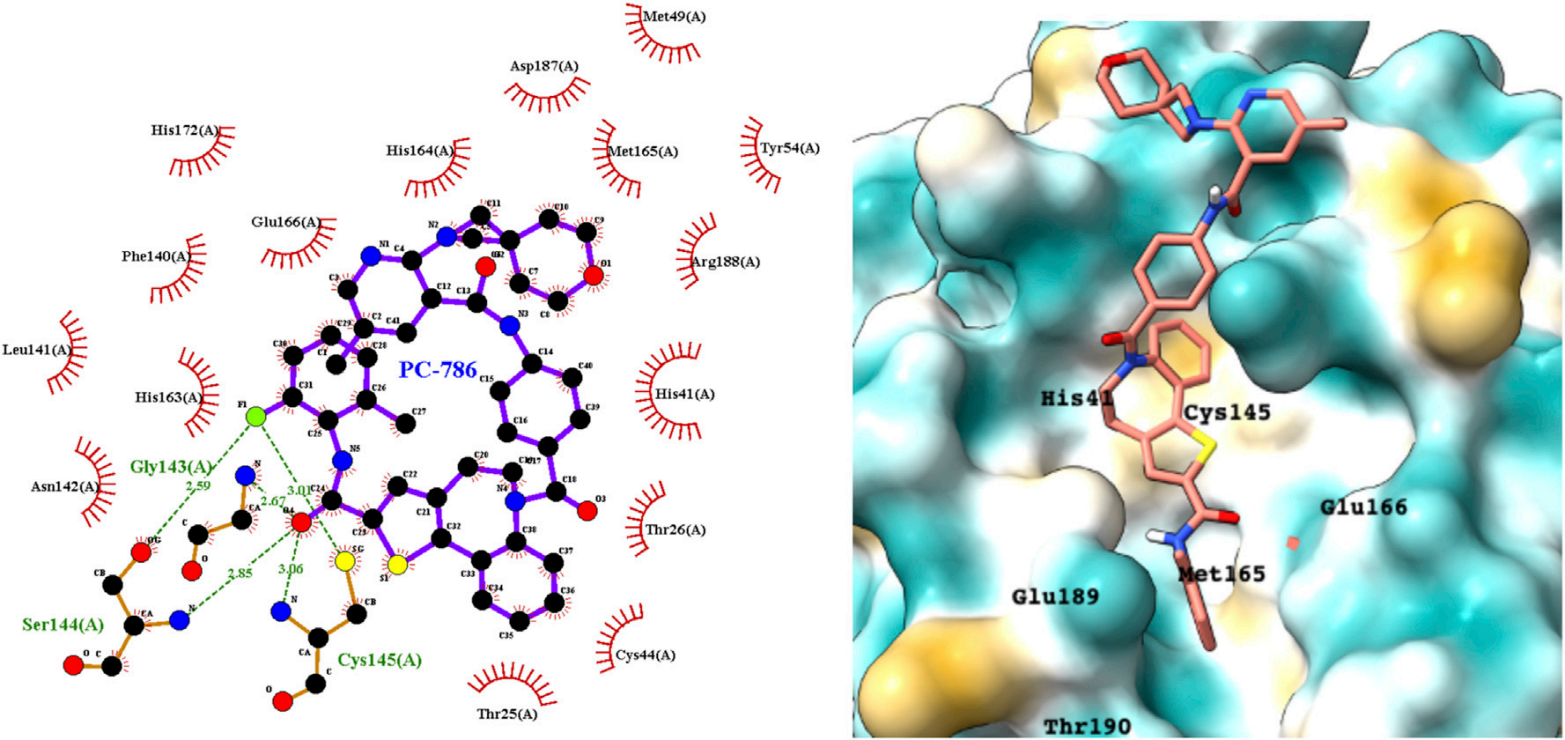
LigPlot (left) and hydrophobic protein surface representation (right) of the main interactions between M^pro^ and PC786. The molecular surface denotes hydrophobicity of the pockets (blue hydrophilic, yellow/brown hydrophobic). Key binding site residues are labeled to help orient the viewer. Other novel putative M^pro^ inhibitors from the short list of 84 drugs.

PC786 targets the respiratory syncytial virus (RSV) L protein and is designed to be a topical inhalation treatment, a likely route of infection for SARS-CoV-2. Figure 5 shows a LigPlot representation of the interactions of key functional groups in PC786 with protease active site residues, with the specific interactions listed in Supplementary Table S2. It forms a hydrogen bond network with Gly143, Ser144, and Cys145. Figure 5 illustrates the binding pose of PC786 in the M^pro^ binding site after MD simulations based on the structure obtained from Vina docking calculations. The hydrophobic phenyl ring of the benzazepine moiety projects into a hydrophobic pocket formed partly by Thr25 and Thr26.

The predicted binding energies of the 84 drugs in the short list are summarized in Supplementary Table S1, along with details of any experiments to determine their activities against M^pro^ or SARS-CoV-2 *in vitro* or *in vivo*. This suggests that our screening and MD simulation methods are sufficiently robust and accurate to identify drugs for repurposing against SARS-CoV-2 and, more broadly, other coronaviruses. The 33% of drugs in the hit list that have not been reported before are clearly of potential interest as novel drugs for treating COVID-19. We discuss below some of the more interesting and novel hit compounds with stronger binding affinities.

**FIGURE 6:**
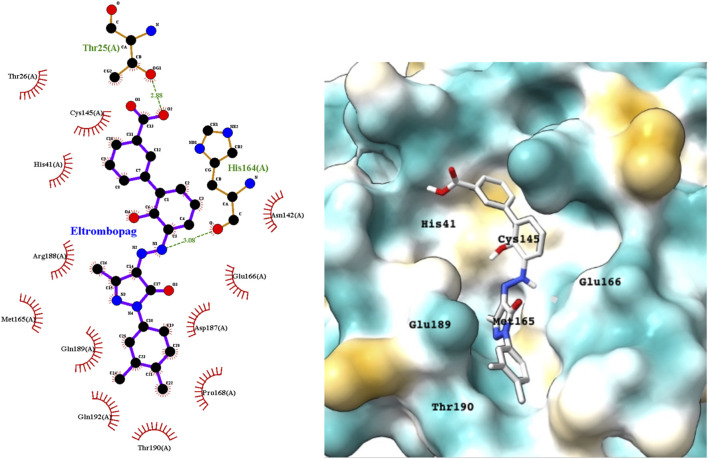
LigPlot (left) and hydrophobic M^pro^ protein surface representation (right) of the main interactions between M^pro^ and eltrombopag and M^pro^. The molecular surface denotes hydrophobicity of the pockets (blue hydrophilic, yellow/brown hydrophobic).

Eltrombopag is a thrombopoietin (TPO) receptor agonist that acts at the transmembrane domain of its cognate receptor C-Mpl *via* a histidine residue that occurs only in humans and apes. It scored highly in the docking studies, suggesting that it could inhibit the M^pro^ and exhibit antiviral activity. Figure 6 shows a LigPlot representation of the interactions of key functional groups in eltrombopag with protease active site residues. The binding pose of eltrombopag in the active site of M^pro^ from the MD simulations is also shown in Figure 6. Close analysis of the binding mode shows that eltrombopag occupies the main part of the M^pro^ binding pocket, with the hydrophilic biphenyl moiety binding to the hydrophobic pocket formed partly by Cys145. The hydrophilic pyrazolone lies in a polar cleft bounded by Glu189 and Glu166, with the terminal dimethyl phenyl ring undergoing a hydrophobic interaction with Thr190.

Eltrombopag is of particular interest as an M^pro^ inhibitor lead because it is novel and is also a member of a large class of small molecular TPO receptor agonists that may also exhibit activity against the viral protease, and potentially the spike protein and human ACE2.(Tarasova and Winkler, 2009). However, given the clotting disorders that SAR-CoV-2 generates, the TPOR agonist activities would need to be minimized to prevent platelet enhancement, while retaining or enhancing the antiviral activities.

Apart from the drugs discussed above, several other drugs in Supplementary Table S1 are of interest. There are several other ergot derivatives with good predicted binding affinities to M^pro^. Metergotamine and dihydroergocristine were predicted to have ΔG_bind_ of –29 and –24 kcal/mol, respectively.

## Discussion

Our virtual screening approach, using Autodock Vina and MD simulation in tandem to calculate binding poses and energies for repurposed drugs, identified 84 compounds with potential for treating COVID-19. The top hits from our study consisted of a mixture of antiviral agents, natural products and drugs developed for other applications and that have additional models of action. We now discuss the results of our computational screening in the context of other computational studies of M^pro^ in the literature.

### Relevant Computational Drug Repurposing Modeling Studies

We reviewed the literature for other *in silico* studies that also identified some of these hit compounds as potential M^pro^ inhibitors and SARS-CoV-2 antiviral agents. Many drugs on the list in Supplementary Table S1 are predicted by published computational studies to be potential inhibitors of SARS-CoV-2 target proteins, largely M^pro^ but also RNA-dependent RNA polymerase (RdRp), spike, helicase, 2′-O-methyltransferase, nsp16/nsp10 complex, nsp1, PL^pro^, nsp3, and nsp12, and human angiotensin converting enzyme 2 (ACE2). Satisfyingly, those with the best predicted binding affinity from our study have also been of greatest interest clinically, with a larger number of *in vitro* assay results and clinical trials for drugs with the highest binding affinities (see below).

#### Modeling Studies Related to the Top 10 Predicted Drugs for Repurposing

Simeprevir was reported to be an inhibitor of the M^pro^ by Abhithaj et al. (2020) They used a pharmacophore search followed by grid-based ligand docking (GLIDE, Schrodinger) and binding energy estimates from the MMGBSA method of −81.7 kcal/mol. However, they did not use MD to simulate the interaction of simeprevir in the M^pro^ binding site. Similarly, sofosbuvir was reported to be a strong inhibitor of the protease by Lo et al. (2021).

The potential protease inhibition properties of lopinavir and ritonavir were reported by Bolcato et al., who used supervised MD to calculate the trajectories of the ligands in the protease binding site (Bolcato et al., 2020). Muralidharan et al. also used AutoDock (another docking program similar to Vina produced by the Scripps group) followed by MD simulations using the Generalized Amber Force Field (GAFF) in Amber16 to screen for repurposed drugs (Muralidharan et al., 2021). They reported AutoDock binding energies for lopinavir, oseltamivir, and ritonavir of −4.1 kcal/mol, −4.65 kcal/mol, and −5.11 kcal/mol, respectively, but did not provide the binding energies from the MD calculations. The best-known antiviral drug, which has been the subject of several clinical trials for COVID-19, is remdesivir (Hendaus, 2021). The potential inhibition of M^pro^ by this drug has been reported in several computational screening studies. For example, Al-Khafaji and colleagues reported a combined computational docking and MD study of a range of antiviral drugs to the viral protease (Al-Khafaji et al., 2021). They calculated a binding energy for remdesivir of −65.19 kcal/mol from a gromacs simulation and a MMGBSA binding energy calculation. Beck et al. reported a K_d_ for binding of remdesivir to 3CLPro of 113 nM using a deep learning model.

Novel potential M^pro^ inhibitors that emerged from our study included the ergot alkaloids ergotamine, mergocriptine, the thrombopoietin receptor agonist eltrombopag (ranked 13 with ΔG_MMPBSA_ = –28.2 kcal/mol, see Supplementary Table S1), bemcentinib, PC786, and montelukast. These drugs were predicted to have better binding energies than the antiviral drugs discussed above and were not previously known to be antiviral.

Gurung et al. reported potential binding of ergotamine to the SARS-CoV-2 main protease in a preprint (Gurung et al., 2020). They employed AutoDock Vina but without subsequent MD simulation of the complex. They reported the binding energy as −9.4 kcal/mol for dihydroergotamine and −9.3 kcal/mol for ergotamine. Mevada et al. also reported *in silico* estimates of the binding of ergotamine to the protease using AutoDock Vina for the virtual screening (Mevada et al., 2020). They found the drug bound with an energy of −10.2 kcal/mol, calculated using Vina (no subsequent MD simulation). Gul et al. used a similar docking approach, this time with MD simulation, and identified ergotamine and its derivatives, dihydroergotamine and bromocriptine, as having high binding affinity to SARS-CoV-2 M^pro^. Ergotamine is an alpha-1 selective adrenergic agonist and vasoconstrictor and exhibited a favorable docking binding energy against SARS-CoV-2 M^pro^ of −8.6 kcal/mol. Dihydroergotamine, the 9,10-alpha-dihydro derivative of ergotamine, showed a similar high affinity of −8.6 kcal/mol, and bromocriptine had a high affinity of −9.2 kcal/mol. Ergotamine has also been predicted to bind tightly to the SARS-CoV-2 spike (S) protein (Qiao et al., 2020).

Montelukast has been shown to inhibit at least one other protease, eosinophil protease (Langlois et al., 2006). Mansoor and colleagues deduced that it may bind to M^pro^ on the basis of a simple molecular docking study (Mansoor et al., 2020). Wu et al. also reported putative binding of montelukast to M^pro^ in a computational study using the same Internal Coordinate Mechanics modeling methods (Wu et al., 2020). No accurate binding affinities were reported in either study.

There is very little published work on the PC786 SARS-CoV-2 efficacy or predicted binding affinity to M^pro^. Panda and coworkers reported a binding energy ΔG_bind_ for PC786 of −179.79, tighter binding than calculated for lopinavir (−131.49 kJ/mol), using a combined docking and MD approach (Panda et al., 2020). Like our study, they employed Autodock Vina to dock a molecular library into the active site of M^pro^, followed by MD simulation using GROMACS.

#### Relevant Modeling Studies of the Drugs From the List of 84 Drugs

Several *in silico* screening studies have identified eltrombopag as a potential SARS-CoV-2 drug. Feng et al.‘s study suggested that eltrombopag bound not only to the M^pro^ active site but also to the viral spike protein and to human ACE2 (Feng et al., 2020). This potential synergistic polypharmacy could be particularly beneficial for treating COVID-19. Eltrombopag has also been proposed as a useful drug against SARS-CoV-2 spike protein on the basis of its predicted strong binding to a pocket in the fusion cores of S2 domain (Feng et al., 2020). Eltrombopag was identified as having a high binding affinity to human ACE2, the primary binding site for the SARS-CoV-2 spike protein. This virtual screening study also used Autodock Vina, but no subsequent MD simulation was used for the top hit compounds from the screen. Surface Plasmon Resonance (SPR) was used to assess the binding of the drug to M^pro^.

Other drugs with binding energies stronger than –25 kcal/mol include galicaftor (in clinical trial for cystic fibrosis), rolitetracycline (a broad spectrum antibiotic), disogluside (a natural product from *Dioscorea nipponica Makino* that reduces liver chronic inflammation and fibrosis), zafirlukast (a leukotriene receptor antagonist for asthma), diosmin (a natural flavone for treating venous disease), AZD-5991 (in clinical trial for relapsed or refractory hematologic malignancies), and ruzasvir (in clinical trials for treatment of hepatitis C). Li et al. also reported predicted M^pro^ binding for galicaftor (Li et al., 2020). These drugs and natural products merit assessment in SAR-CoV-2 assays and M^pro^ inhibition experiments.

As we stated in the introduction, few studies have used computational docking followed by MD simulation of the best repurposing candidates, and fewer still have reported experimental validation of the computational predictions. Here, we report a comprehensive review of experimentally determined protein target, *in vitro*, or *in vivo* activities of our 84 top binding drug candidates.

### Experimental Validation of Biological Activity of Computational Repurposing Candidates

Clearly, blind computational predictions of likely M^pro^ activity and, potentially, SARS-CoV-2 activities are of limited use if the predictions are not validated experimentally. Supplementary Table S1 shows that 70% of the top 10 hit compounds have confirmed experimental activity against SARS-CoV-2, and 25% of the 84 entries in this table also have confirmed experimental activity either against SARS-CoV-2 or M^pro^. Of the remaining 75% of putative repurposed drugs, most have not been studied experimentally so they may have relevant antiviral activity, at least *in vitro*. The relatively high experimental validation rate of compounds predicted to be strong binders to M^pro^ suggests that our computational paradigm is useful for selecting drugs for repurposing against SARS-CoV-2. Clearly, computational studies are investigating molecular interactions at the target, which will be most useful for identifying candidates with strong target activity, M^pro^ in this instance. The high experimental validation rate also strongly suggests that the drugs not yet experimentally tested should at least be screened in an *in vitro* antiviral assay.

#### Validation of SARS-CoV-2 Activity of Top 10 Predicted Drugs for Repurposing

**FIGURE 7:**
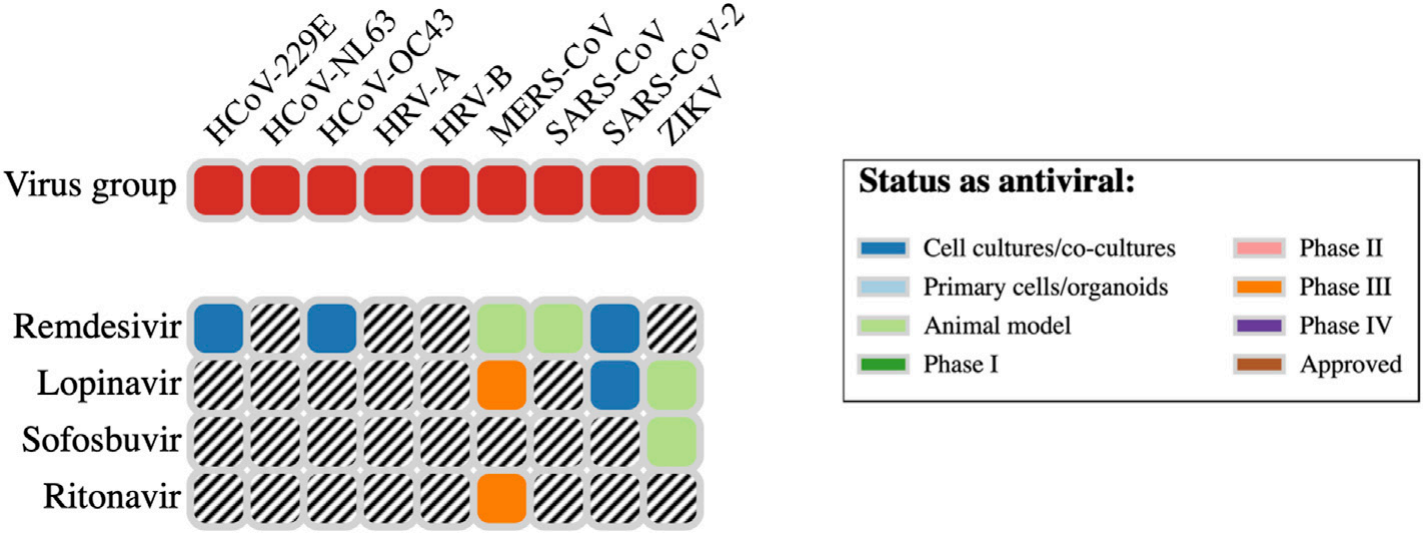
Spectrum of antiviral activity and nature of assessment for four antiviral hit drugs.

The website DrugVirus.info provides a concise picture of the broad-spectrum antiviral activity of a range of drugs. A summary for four of the top 10 antiviral hits (Table 1) from our *in silico* screens is provided in Figure 7. Here, we discuss the experimental SARS-CoV-2 or molecular target activity of seven of the top ten repurposing drug candidates identified by our computational studies—bemcentinib, montelukast, simeprevir, sofosbuvir, lopinavir, ritonavir, and remdesivir.

Bemcentinib selectively inhibits AXL kinase activity, which blocks viral entry and enhances the antiviral type I interferon response. Its *in vitro* activity against SARS-CoV-2 has been assessed by several groups. In a Vero cell assay, Liu et al. reported 10–40% protection at 50 µM (Liu et al., 2020). However, in an alternative assay using human Huh7.5 cells (Dittmar et al., 2021), bemcentinib exhibited an IC_50_ of 100 nM and CC_50_ of 4.7 µM. These authors also developed an assay in Vero cells and reported an IC_50_ of 470 nM and CC_50_ of 1.6 µM, considerably higher activity than that reported by Liu et al. As a result, it is an investigational treatment for COVID-19 (www.clinicaltrialsregister.eu), with a phase 2 trial underway (Wilkinson et al., 2020). Dittmar and co-workers also reported an ED_50_ for bemcentinib of 0.1 µM (Huh7.5 cells), 0.47 µM (Vero cells), and 2.1 µM (Calu3 cells) (Dittmar et al., 2021). Six of the top 10 drugs (Table 1) are antiviral agents. Using a Vero E6 cellular infection model, they also reported that simeprevir was the only drug among their prioritized candidates that suppressed SARS-CoV-2 replication at below 10 μM. Dose–response studies showed that simeprevir had an EC_50_ of 4 μM and a CC_50_ of 20 μM, similar to remdesivir in their experiments. Simeprevir had an experimental *in vitro* EC_50_ activity of 4.08 μM. Ma et al. developed a fluorescence resonance energy transfer (FRET)-based enzymatic assay for the SARS-CoV-2 M^pro^ and used it to screen a library of M^pro^ inhibitors (Ma et al., 2020). In their assay, simeprevir exhibited an IC_50_ of 14 ± 3 µM.

Sofosbuvir was reported to be a strong inhibitor of the main protease by Lo et al. (2021). It has *in vitro* antiviral EC_50_ values of 6.2 and 9.5 μM (Sacramento et al., 2021). Lopinavir exhibits an antiviral *in vitro* EC_50_ of 5.7 µM in one study (Yamamoto et al., 2020), and an EC_50_ of 26.6 μM in another study (Choy et al., 2020). It is also the subject of multiple single-agent and combination human trials (e.g., Cao et al., 2020; Costanzo et al., 2020). Ritonavir has an experimental *in vitro* EC_50_ of 8.6 µM (Yamamoto et al., 2020), and it too is being assessed in multiple single-agent and combination human trials (e.g., Cao et al., 2020; Verdugo-Paiva et al., 2020). Costanzo and colleagues likewise reported high protease binding for these two antiviral drugs (Costanzo et al., 2020). They also reported updates on experimental drugs successfully employed in the treatment of the disease caused by SARS-CoV-2 coronavirus. Patient recovery has been reported after treatment with lopinavir/ritonavir (used to treat HIV infection) in combination with the anti-flu drug oseltamivir.

Remdesivir has also been assessed in multiple human trials (e.g., Wang Y. et al., 2020; Olender et al., 2021), and it has reported antiviral *in vitro* EC_50_ values of 23.2 μM (Choy et al., 2020) and 0.77 μM and a CC_50_ > 100 μM in another study (Wang M. et al., 2020). It had a SARS-CoV-2 EC_50_ in Vero cells of 6.6 μM and CC_50_ > 100 µM (Pirzada et al., 2021). Beck et al. reported a K_d_ for binding of remdesivir to M^pro^ of 113 nM using a deep learning model. Liu et al. reported an *in vitro* assay that exploited the pronounced cytopathic effects of SAR-CoV-2 on Vero cells and the ability of a range of antiviral drugs to protect cells against the virus (Liu et al., 2020). In their assay, remdesivir exhibited an IC_50_ of 2.5 µM and a CC_50_ of 175 µM, while sofosbuvir, lopinavir, and ritonavir were inactive.

Montelukast has been shown to produce a significant reduction in SARS-CoV-2 infection in the treated elderly asthmatic patients. Kumar et al. also reported *in vitro* SARS-CoV-2 inhibition, with an IC_50_ of 18.8 µM and CC_50_ > 20 µM (Bozek and Winterstein, 2020; Kumar et al., 2021).

#### Other Novel Putative SARS-CoV-2 Drugs From the List of 84 Drugs

We also highlight some novel and interesting drugs for repurposing in the list of 74 (Supplementary Table S1) with weaker predicted binding affinity than the top 10 listed in Table 1. As stated above, 25% of the drugs in Supplementary Table S1 have reported experimental data that support the validity of predictions from our computational experiments.

Eltrombopag, a thrombopoietin receptor agonist used to treat thrombocytopenia, has a reported IC_50_ 8.3 µM for SARS-CoV-2 infection in Vero and Calu-3 cells (Ko et al., 2021). Recently, Vogel et al. reported direct inhibition of cytomegalovirus (CMV) by therapeutic doses of eltrombopag used to treat thrombocytopenia (Vogel et al., 2019). They showed that eltrombopag inhibits the late stages of the HCMV replication cycle and reduces virus titers by 1.8 × 10^4^-fold at 10 µM and by 15-fold at 500 nM.

Saquinavir, an HIV protease inhibitor used in combination with other antiretroviral agents for the treatment of HIV-1, displays an *in vitro* EC_50_ of 8.8 µM (Yamamoto et al., 2020); zafirlukast, a leukotriene receptor antagonist used for prophylaxis and chronic treatment of asthma, exhibits an *in vitro* SARS-CoV-2 IC_50_ value of 3.6 µM (Zeng et al., 2021). Zhu and coworkers also measured the SARS-CoV-2 and M^pro^ inhibition of zafirlukast (Zhu et al., 2020). The IC_50_ for M^pro^ was 24 µM and the EC_50_ for the virus >20 µM.

Eravacycline, a tetracycline antibiotic used to treat complicated intra-abdominal infections, has a reported *in vitro* activity against recombinant SARS-CoV-2, SARS-CoV, and MERS-CoV main proteases, with IC_50_ values of 1.7, 10.0, and 16.4 µM, respectively. It also inhibits SARS-CoV-2 infection in VeroE6 cells with an IC_50_ = 30.6 µM (Reig and Shin, 2020). Umifenovir (Arbidol), exerts antiviral effects through multiple pathways that see its use against a variety of enveloped and non-enveloped RNA and DNA viruses. It inhibits coronavirus OC43 with an IC_50_ of 4.4 µM and SARS-CoV-2 with an IC_50_ of 10 µM (Xiao et al., 2020). It has also been reported to inhibit SARS-CoV-2 infection at 10–30 μM *in vitro* (Vafaei et al., 2020). Multiple clinical trials show a larger negative rate of PCR on day 14 in adult COVID-19 patients (Huang et al., 2021), and it shortens the viral shedding interval (Huang et al., 2020). Atazanavir, an antiretroviral drug of the protease inhibitor (PI) class, displays SARS-CoV-2 inhibition (in Vero cells) with an EC_50_ of 2 μM and a CC_50_ of 312 µM. It is also active against SARS-CoV-2 infection in a human epithelial pulmonary cell line (A549) with an EC_50_ of 0.22 µM (Fintelman-Rodrigues et al., 2020). Zhu and corkers measured the SARs-CoV-2 and M^pro^ inhibition of zafirlukast (Zhu et al., 2020). The IC_50_ for M^pro^ was 24 µM and the EC_50_ for the virus is >20 µM.

The protease binding of rolitetracycline has been reported by Durdagi (2020) and Gul et al. (2020) The potential of the natural product diosmin as an antiviral agent targeting M^pro^ has also been reported in several recent computational studies (Arun et al., 2020; Ngo et al., 2020; Peterson, 2020b; Peterson, 2020a). Chakraborti et al. reported the potential of ruzasvir as a drug against SARS-CoV-2, although no data were provided (Chakraborti et al., 2020).

The prognostic value of our computational approach has been demonstrated by the fact that it identified a diverse range of drugs that have been reported in other computational studies or that exhibit useful SARS-CoV-2 antiviral effects *in vitro*. The antiviral drugs simeprevir, sofosbuvir, lopinavir, ritonavir, and remdesivir exhibit strong antiviral properties, and several are in clinical trial or used against SARS-CoV-2. These drugs have also been reported as binding to M^pro^ by numerous virtual screening studies, and by *in vitro* assays. The more interesting and least studied hit drugs among our candidate list, bemcentinib, PC786, montelukast, ergotamine, and mergocriptine, were predicted to have binding affinities equal to or greater than the antiviral drugs, and have also been shown to have *in vitro* antiviral activity against SARS-CoV-2. A few computational studies mostly using less rigorous methods than those employed here have also suggested that these drugs may bind to M^pro^.

This high validation success rate strongly suggests that this type of virtual screening approach is capable of identifying compounds with potentially useful activity against SARS-CoV-2 and, by analogy, other coronaviruses. In particular, the 28 drugs for which no SARS-CoV-2 activity has been yet reported may be of particular interest for *in vitro* screening. The results of the current drug repurposing study provide information that could be useful to identify additional candidate drugs for testing for use in the current pandemic, as well as a rational computational paradigm for identifying therapeutic agents for future viral pandemics.

## Materials and Methods

Protein Structure Preparation and Grid Preparation

The crystal structure of the COVID-19 M^pro^ was downloaded from the RCSB PDB (http://www.rcsb.org; refcode 6Y2F) (Zhang L. et al., 2020).

Protein preparation and removal of non-essential and non-bridging water molecules for docking studies were performed using the UCSF Chimera package (https://www.cgl.ucsf.edu/chimera/) (Pettersen et al., 2004) AutoDock Tools (ADT) software was used to prepare the required files for Autodock Vina by assigning hydrogen polarities, calculating Gasteiger charges to protein structures and converting protein structures from the pdb file format to pdbqt format (Forli et al., 2016). The surface area of the 3CLPro binding pocket is 335 Å^2^, and the volume is 364.101 Å^3^ (Tian et al., 2018).

As recommended by Llanos et al., the ability of Vina to redock known ligands from x-ray structures was assessed to determine the reliability of the algorithm for this target. Table 2 shows the RMSD values for redocking the ligands for 10 experimental structures of M^pro^ with bound ligands. The relatively low RMSD values show that Vina can recapitulate the experimental binding poses well.

**TABLE 2:** RMSD errors for redocking small molecule ligands into the binding site of M^pro^.

Crystal ligand	Docking score (kcal/mol)	RMSD (Å)
5R7Y	−5.0	0.44
5R81	−5.0	0.51
5RE4	−4.2	0.62
5REJ	−5.3	0.52
5RG0	−4.5	0.55
6LU7	−6.6	0.44
6W63	−7.2	0.39
5R7Z	−5.3	0.57
5REL	−5.6	0.51
5R83	−5.4	0.57

Screening Databases

Drugs were downloaded from the DrugBank database (Wishart et al., 2018) and CHEMBL database (FDA approved) (Gaulton et al., 2017). A total of 8,773 and 13,308 drugs were retrieved from DrugBank and CHEMBL database, respectively. The drugs were downloaded in sdf format and converted to pdbqt format using Raccoon (Forli et al., 2016).

Docking Methodology Small-molecule ligand structures were docked against protein structure using the AutoDock Vina (version 1.1.3) package (Forli et al., 2016). AutoDock Vina employs gradient-based conformational search approach and an energy-based empirical scoring function that includes an approximate correction for ligand conformational entropy. AutoDock Vina is also flexible, easily scripted, and extensively validated in many published studies with a variety of proteins and ligands and takes advantage of large multi-CPU or -GPU machines to run many calculations in parallel. The code has also been employed very successfully to dock millions of small-molecule drug candidates into a series of protein targets to discover new potent drug leads. The package includes useful scripts for generating modified pdb files required for grid calculations and for setting up the grid calculations around each protein automatically. The software requires the removal of hydrogens, addition of polar hydrogens, setting of the correct atom types, and calculation of atom charges compatible with the AutoGrid code. The algorithm generates a grid around each protein and calculates the interaction energy of a probe noble gas atom at each grid position outside and within internal cavities of the protein. The grid resolution was set to 1 Å, the maximum number of binding modes to output was fixed at 10, and the exhaustiveness level (controlling the number of independent runs performed) was set at 8. The docking employed a genetic algorithm to optimize the binding conformations of the ligands during docking to the protease site. Drugs were docked individually to the active site of M^pro^ (3CLPro, refcode 6Y2F) with the grid coordinates (grid center) and grid boxes of appropriate sizes generated by the bash script vina_screen.sh (Supplementary Information). The top scored compounds were identified with a python script 1 py (Supplementary Information) and subjected to molecular dynamic simulation. The docked structures were analyzed using UCSF Chimera (Pettersen et al., 2004) and LigPlot + software (Laskowski and Swindells, 2011) to illustrate hydrogen-bond and hydrophobic interactions. A total of fifty top compounds were selected from each of the DrugBank and CHEMBL compounds. Sixteen compounds were common to both database top hits. Molecular dynamics studies were conducted on the unique set of eighty-four compounds from both sets.

Molecular Dynamics Simulation The top screened compound complexes with protease were minimized with CHARMm force field. The topology files of the ligands were prepared from Swissparam (http://www.swissparam.ch/) (Zoete et al., 2011) and minimized in Gromacs 2020 (http://www.gromacs.org/) (Abraham et al., 2015). Docked complexes of ligands and COVID-19 M^pro^ protein were used as starting geometries for MD simulations. Simulations were carried out using the GPU accelerated version of the program with the CHARMm force field I periodic boundary conditions in ORACLE server. Docked complexes were immersed in a truncated octahedron box of TIP3P water molecules. The solvated box was further neutralized with Na + or Cl− counter ions using the tleap program. Particle Mesh Ewald (PME) was employed to calculate the long-range electrostatic interactions. The cutoff distance for the long-range van der Waals (VDW) energy term was 12.0 Å. The whole system was minimized without any restraint. The above steps applied 2,500 cycles of steepest descent minimization followed by 5,000 cycles of conjugate gradient minimization. After equilibration at 300 K using Langevin thermostat NVT ensemble for 50 ps, the system was then equilibrated at 1 atm pressure using Berendsen thermostat NPT ensemble for 50 ps. After the system was fully equilibrated at the desired temperature and pressure (NVT/NPT ensembles), we used Parrinelo–Rahman pressure coupling to run MD for data collection. Duplicate production runs starting with different random seeds were also run to allow estimates of binding energy uncertainties to be determined. Finally, a production run of 20 ns of MD simulation was performed.

During the MD procedure, the SHAKE algorithm was applied for the constraint of all covalent bonds involving hydrogen atoms. The time step was set to 2 fs. The structural stability of the complex was monitored by the RMSD and RMSF values of the backbone atoms of the entire protein. Calculations were also performed for up to 100 ns on few compounds to ensure that 20 ns is sufficiently long for convergence. We checked the RMSD of M^Pro^ and drug during this time and it was within the range of 1.5 Å. The RMSF graph revealed minimal fluctuations and relatively stable conformations of SARS-CoV-2 M^pro^ bound to screened drugs.

The protein–ligand binding affinities were evaluated in two ways. One calculates the energies of solvated SARS-CoV-2 protease and small-molecule ligands and the other calculates that of the bound complex and derive the binding energy by subtraction.

ΔE(bind)=ΔE(complex)-(ΔE(protein)+ΔE(ligand)) (1)

We also calculated binding free energies using the molecular mechanics Poisson Boltzmann surface area (MM/PBSA) tool in GROMACS that uses the nonbonded interaction energies of the complex. The method is also a widely used method for binding free energy calculations (Spiliotopoulos et al., 2012). However, accurate calculation of absolute binding energies requires very extensive sampling, so the methods we employed provide accurate relative binding energies of ligands that are useful for ranking them, as we have done in this work.

We used GMXPBSA2.1 program to perform MM/PBSA calculations on selected docked complexes derived from GROMACS trajectories (Paissoni et al., 2015). It is a suite of Bash/Perl scripts for streamlining MM/PBSA calculations on structural ensembles derived from GROMACS trajectories and to automatically calculate binding free energies for protein–protein or ligand–protein. GMXPBSA 2.1, which provides the freedom to calculate free binding energy of complexes with any force field, calculates diverse MM/PBSA energy contributions from molecular mechanics (MM) and electrostatic contribution to solvation (PB) and non-polar contribution to solvation (SA). This tool combines the capability of MD simulations (GROMACS) and the Poisson–Boltzmann equation (APBS) for calculating solvation energy (Baker et., 2001). The g_mmpbsa tool in GROMACS was used after molecular dynamics simulations, and the output files obtained were used to post-process binding free energies by the single-trajectory MMPBSA method. In the current study, we considered 100 frames at equal distance from 20-ns trajectory files.

Specifically, for a non-covalent binding interaction in the aqueous phase, the binding free energy, ΔG (bind,aq), is:

ΔG(bind,aqu)=ΔG(bind,vac)+ΔG(bind,solv) (2)

where ΔG (bind, vac) is the binding free energy in vacuum, and ΔG (bind, solv) is the solvation free energy change upon binding:

ΔG(bind,solv)=ΔG(R:L,solv)-ΔG(R,solv)-ΔG(L,solv) (3)

where ΔG (R:L,solv), ΔG (R,solv), and ΔG (L,solv) are solvation free energies of complex, receptor, and ligand, respectively.

Method Note Added in Proof

Guterres and Im recently showed how substantial improvements in protein–ligand docking results could be achieved using high-throughput MD simulations (Guterres and Im, 2020). As with our study, they also employed AutoDock Vina for docking, followed by MD simulation using CHARMM. The MD parameters they advocated were very similar to those used in our study. Proteins were solvated in a box of TIP3P water molecules extending 10 Å beyond the proteins and the particle-mesh Ewald method was used for electrostatic interactions. Nonbonded interactions over 10 and 12 Å were truncated. Their systems were minimized for 5,000 steps using the steepest descent method followed by 1 ns of equilibration with an NVT setting. For each protein–ligand complex, they ran 3 × 100-ns production runs from the same initial structure using different initial velocity random seeds and an integration step size of 2 fs. Over 56 protein targets (of seven different protein classes) and 560 ligands, this shows 22% improvement in the area under the receiver operating characteristic curve, from an initial value of 0.68 using AutoDock Vina alone to a final value of 0.83 when the Vina results were refined by MD.

## Data Availability

The data have been deposited in the OPAL repository at La Trobe University and are available at DOI 10.26181/19235004.
