# Supplementary material for: Computational Repurposing of Drugs and Natural Products Against SARS-CoV-2 Main Protease (Mpro) as Potential COVID-19 Therapies
Source: Front Mol Biosci. 2022 Mar 14;9:781039. doi: 10.3389/fmolb.2022.781039 (PMC8964187; doi:10.3389/fmolb.2022.781039)
Supplement: Supplementary file 1 [file DataSheet1.PDF]

Computational screening of repurposed drugs and natural products against SARS-CoV-2 main protease (M^{pro}) as potential COVID-19 therapies

Sakshi Piplani[1-2], Puneet Singh[1-2], Nikolai Petrovsky[1-2], David A. Winkler[3-5]

[1] College of Medicine and Public Health, Flinders University, Bedford Park 5046, Australia

[2] Vaxine Pty Ltd, 11 Walkley Avenue, Warradale 5046, Australia

[3] La Trobe University, Kingsbury Drive, Bundoora 3042, Australia

[4] Monash Institute of Pharmaceutical Sciences, Monash University, Parkville 3052, Australia

[5] School of Pharmacy, University of Nottingham, Nottingham NG7 2RD. UK

Supplementary information

Table S1. Binding energies and published SARS-Cov-2 data for 84 top ranked small molecule ligands

	Name	ChEMBL (C)) or Drugbank (D) ID	ΔG_{MMPBSA} kcal/mol	SARS-Cov-2 data
1	Bemcentinib	C 3809489	-33.9	Phase 2 clinical trial,(Wilkinson et al., 2020) ED_{50} 0.1 (Huh7.5), 0.47 (Vero), 2.1 (Calu3) µM,(Dittmar et al., 2020) predicted 2'-O-methyltransferase nsp16/nsp10 complex binding(Encinar and Menendez, 2020)
2	PC786	C 4291143	-33.0	Predicted spike glycoprotein,(Allam et al., 2020) M^{pro}, and ACE2 binding(Panda et al., 2020)
3	Montelukast	C 787	-32.6	Significant reduction in SARS-CoV-2 infection in elderly asthmatic patients treated with MK.(Bozek and Winterstein, 2020) SARS-CoV-2 inhibition IC_{50} 18.8 µM, CC_{50} >20 µM.(Kumar et al., 2021) Several predicted M^{pro} binding studies e.g.(Bozek and Winterstein, 2020;Copertino et al., 2021)
4	Ergotamine	C 442	-31.6	Several predicted M^{pro} binding studies e.g.(Gul et al., 2020;Gurung et al., 2020;Qiao et al., 2020)
5	Simeprevir	D06290	-31.5	In vitro EC_{50} 4.08 µM, and many predicted M^{pro} binding studies e.g.(Lo et al., 2020), many predicted Mpro binding studies e.g.(Alamri et al., 2020;Hakmi et al., 2020;Hosseini et al., 2020), predicted RdRp binding(Athar and Beg, 2020;Cozac et al., 2020)
6	Sofosbuvir	D08934	-31.0	In vitro EC_{50} values of 6.2 and 9.5 µM.(Sacramento et al., 2020) Predicted RdRp(Elfiky et al., 2020;Jacome et al., 2020) binding
7	Lopinavir	D01601	-30.7	In vitro EC_{50} 5.73 µM,(Yamamoto et al., 2020) Multiple single agent and combination human trials e.g.(Cao et al., 2020;Costanzo et al., 2020). In vitro EC_{50} 26.63 µM.(Choy et al., 2020) Predicted M^{pro} binding(Bolcato et al., 2020;Chtita et al., 2021;Muralidharan et al., 2021)

	Name	ChEMBL (C)) or Drugbank (D) ID	ΔG_{MMPBSA} kcal/mol	SARS-Cov-2 data
8	Ritonavir	D00503	-30.2	In vitro EC_{50} 8.63 μM,(Yamamoto et al., 2020) Multiple single agent and combination human trials e.g.(Cao et al., 2020;Verdugo-Paiva et al., 2020) Predicted M^{pro} (Muralidharan et al., 2021) helicase and RdRp binding(Beck et al., 2020)
9	Mergocriptine	C2105887	-30.1	Predicted 2′-O-ribose methyltransferase(Jiang et al., 2020) binding
10	Remdesivir	D14761	-29.9	Multiple human trials e.g.(Olender et al., 2020;Wang et al., 2020b), in vitro EC_{50} 23.15 μM,(Choy et al., 2020) EC_{50} = 0.77 μM; CC_{50} > 100 μM,(Wang et al., 2020a) SARS-CoV-2 EC_{50} in Vero cells = 6.6 μM, CC_{50} > 100 μM,(Pirzada et al., 2021) and predicted M^{pro} (Rehman et al., 2020) and RdRp(Elfiky, 2020) binding
11	Metergotamine	C2106428	-29.1	Predicted 2′-O-ribose methyltransferase(Jiang et al., 2020) binding
12	Galicaftor	D14894	-28.4	Predicted M^{pro} binding(Li et al., 2020)
13	Eltrombopag	C461101	-28.2	IC_{50} 8.3 μM in Vero and Calu-3 cells,(Ko et al., 2021) Predicted spike(Feng et al., 2020) and RdRp(Gul et al., 2020) binding
14	Saquinavir	C114	-27.9	In vitro EC_{50} 8.83 μM,(Yamamoto et al., 2020) predicted ACE2, M^{pro},(Qiao et al., 2020) and RdRp(Ruan et al., 2021) binding(Alexpandi et al., 2020)
15	Rolitetracycline	C 1237046	-27.6	Predicted M^{pro} (Durdagi et al., 2020) and spike(Senathilake et al., 2020) binding
16	Disogluside	C395414	-27.3	Predicted M^{pro} (Jiang et al., 2020) binding
17	Zafirlukast	D00549	-26.7	In vitro SARS-CoV-2 IC_{50} = 3.6 μM,(Zeng et al., 2021) Predicted spike,(Qiao et al., 2020) M^{pro},(Subramanian, 2020) and 2′-O-ribose methyltransferase(Sharma et al., 2020) binding
18	Diosmin	D08995	-25.8	Predicted M^{pro} (Adem et al., 2020;Peterson, 2020) binding
19	AZD-5991	D14792	-25.2	…
20	Ruzasvir	C 3833385	-25.1	Predicted M^{pro} (Chakraborti et al., 2020) and RdRp(Cozac et al.,

	Name	ChEMBL (C)) or Drugbank (D) ID	ΔG_{MMPBSA} kcal/mol	SARS-Cov-2 data
				2020) binding
21	Rebastinib	C1738757	-24.3	Predicted 2'-O-ribose methyltransferase nsp16 binding(Jiang et al., 2020)
22	RSV-604	D15197	-24.3	…
23	Eravacycline	D12329	-24.2	In vitro against recombinant SARS-CoV-2, SARS-CoV and MERS-CoV 3CL proteases, with IC_{50} of 1.7, 10.0 and 16.4 µM respectively and inhibits SARS-CoV-2 infection of VeroE6 with IC_{50} = 30.6 µM.(Reig and Shin, 2020) Predicted M^{pro} (Wang, 2020;Kouznetsova et al., 2021) binding
24	Lifitegrast	C2048028	-24.1	SARS-CoV-2 spike protein K_D of 1.9 nM and elimination of 99.8% of the RBD-ACE2 protein-protein interaction in SPR competition assay. Blocks SARS-CoV-2 in vitro infection potency of 1.3 mM with CC_{50} > 5mM.(Day et al., 2021) Predicted nsp16/nsp10 complex(Encinar and Menendez, 2020) and RdRp(Cozac et al., 2020) binding
25	10-Deoxymethynolide	D07703	-24.1	…
26	Ledipasvir	D09027	-23.8	SARS-CoV-2 inhibition in Vero E6 cells with EC_{50} = 34.6 µM, CC_{50} > 100 µM.(Pirzada et al., 2021) Predicted M^{pro} binding(Chen et al., 2020)
27	Deldeprevir	C3040582	-23.8	Predicted M^{pro} binding(Hakmi et al., 2020)
28	Rifamycin	D11753	-23.8	…
29	Ethoxazorutoside	C 2106047	-23.8	…
30	Dihydroergocristine	C601773	-23.8	Predicted M^{pro} (Chen et al., 2020) binding
31	Gedatolisib	C592445	-23.8	…
32	Lorecivivint	D14883	-23.8	Predicted M^{pro} (Peterson, 2020) and spike(Panda et al., 2020) binding
33	MK-6325	C4297304	-23.6	…
34	Laniquidar	C539378	-23.1	Predicted RdRp(Arul et al., 2020) and spike(Romeo et al., 2020) binding

	Name	ChEMBL (C)) or Drugbank (D) ID	ΔG_{MMPBSA} kcal/mol	SARS-Cov-2 data
35	Tirabrutinib	C 4071161	-22.9	EC_{50} >10μM in SARS-CoV-2-Nluc neutralization assay(Xie et al., 2020)
36	3-(2-aminoquinazolin-6-yl)-4-methyl-N-[3-(trifluoromethyl)phenyl]benzamide	D06925	-22.9	…
37	Ensartinib	D14860	-22.8	…
38	Anacetrapib	C1800807	-22.4	…
39	Pazinaclone	C2107504	-22.3	Predicted M^{pro} binding(Sobeh et al.)
40	BMS-986142	D15291	-22.2	…
41	Phthalocyanine	D12983	-22.2	Pentalysine β- carbonylphthalocyanine zinc (ZnPc5K) and core chlorin e6 (ce6) SARS-CoV-2 EC_{50} values of 177 nM and 156 nM, and EC_{90} values of 308 nM and 352 nM in Vero cells (UV illumination).(Yu et al., 2021) Predicted nsp1,(de Lima Menezes and da Silva, 2020) M^{pro},(Li et al., 2020) spike,(Romeo et al., 2020) nsp1,(de Lima Menezes and da Silva, 2020) and 2′-O-methyltransferase(Encinar and Menendez, 2020) binding
42	Umbralisib	C3948730	-21.9	…
43	DNK333	C105060	-21.9	…
44	Midostaurin	C608533	-21.7	Predicted M^{pro} binding.(Subramanian, 2020)
45	Umifenovir (Arbidol)	D13609	-21.6	Inhibits coronavirus OC43 with IC_{50} 4.43 μM and SARS-CoV-2 IC_{50} 10 μM.(Xiao et al., 2020) Inhibits SARS-CoV-2 infection at 10-30 μM in vitro.(Vafaei et al., 2020) Multiple clinical trials only show higher negative rate of PCR on day 14 in adult COVID-19 patients.(Huang et al., 2021) Shortens the viral shedding interval.(Huang et al., 2020) Predicted M^{pro} binding.(Naveen and Reddy, 2020)
46	Lumacaftor	D09280	-21.5	Predicted M^{pro} (Alméciga-Díaz et al., 2020;Arul et al., 2020;Chen et al., 2020) and RdRp(Khater et al., 2020) binding
47	TU-100	D12467	-21.3	…

	Name	ChEMBL (C)) or Drugbank (D) ID	ΔG_{MMPBSA} kcal/mol	SARS-Cov-2 data
48	Triamcinolone furetonide	C2105791	-21.2	…
49	Zoliflodacin	C3544978	-21.2	Predicted M^{pro} (Chakraborti and Srinivasan, 2020) and PL^{pro}(Arul et al., 2020) binding
50	KPT-9274	C4297467	-20.9	…
51	Atazanavir	D01072	-20.8	SARS-CoV-2 inhibition (Vero cells) EC_{50} = 2 μM, CC_{50} = 312 μM, and in human epithelial pulmonary cell line (A549) EC_{50} = 0.22 μM.(Fintelman-Rodrigues et al., 2020) Inhibits SARS-CoV-2 replication, predicted M^{pro} (Fintelman-Rodrigues et al., 2020) and helicase(Borgio et al., 2020) binding
52	Mitratapide	C2104975	-20.8	Predicted 2'-O-ribose methyl-transferase nsp16 binding(Jiang et al., 2020)
53	Tarloxotinib	D14944	-20.5	…
54	Spergualin	C1765508	-20.5	…
55	Moxidectin	D11431	-20.5	SARS-CoV-2 in vitro EC_{50} = 3.1 μM and CC_{50} 6.9 μM.(Jan et al., 2021)
56	PRI-724	D15034	-20.0	…
57	2-[3-(methyl[1-(2-naphthoyl)piperidin-4-yl] amino}carbonyl)-2-naphthyl]-1-(1-naphthyl)-2-oxoethyl phosphonic acid	D04016	-19.8	…
58	ASP-4058	D11819	-19.8	…
59	Beclabuvir	DB12225	-19.7	Modest in vitro SARS-CoV-2 inhibition at 1 μM and 10 μM.(Massignan et al., 2021) Predicted RdRp(Athar and Beg, 2020) and M^{pro} (Sekhar, 2020) binding
60	Ubrogepant	C2364638	-19.5	Predicted to disrupt spike-ACE2 interaction(Omotuyi et al., 2020)
61	Dihydroergotamine	D00320	-19.5	Inhibits coronavirus OC43 with IC_{50} 5.4 μM, CC_{50} >30 μM.(Xiao et al., 2020) Predicted M^{pro} (Gul et al., 2020;Gurung et al., 2020) and 2′-O-ribose methyltransferase(Sharma et al., 2020)

	Name	ChEMBL (C)) or Drugbank (D) ID	ΔG_{MMPBSA} kcal/mol	SARS-Cov-2 data
				binding
62	Lifirafenib	C 4209157	-19.3	Predicted spike(Arul et al., 2020) and nsp10– nsp16 complex(Encinar and Menendez, 2020) binding
63	Golvatinib	D11977	-18.8	Predicted M^{pro} (Arul et al., 2020) and RdRp(Ruan et al., 2021) binding
64	Tirilazad	D13050	-18.6	Predicted M^{pro} (Sekhar, 2020;Chtita et al., 2021) and nsp1(de Lima Menezes and da Silva, 2020) binding
65	4-[(10s,14s,18s)-18-(2-amino-2-oxoethyl)-14-(1-naphthylmethyl)-8,17,20-trioxo-7,16,19-triaza spiro[5.14]icos-11-en-10-yl]benzylphosphonic acid	D03276	-18.6	…
66	Etamocycline	C3989417	-16.1	
67	Quarfloxin	C3989407	-15.9	Predicted spike,(Romeo et al., 2020) PL^{pro}(Arul et al., 2020) and M^{pro} (Alexpandi et al., 2020)
68	2'-(4-dimethylamino-phenyl)-5-(4-methyl-1-piperazinyl)-2,5'-bi-benzimidazole	D04011	-15.7	…
69	Dihydrostreptomycin	C 1950576	-15.6	Predicted nsp3 and nsp10– nsp16 complex binding(Kandwal and Fayne, 2020)
70	Rimegepant	C2178422	-15.6	…
71	Bezitramide	C2104149	-15.4	…
72	Flutroline	C57241	-15.3	Predicted 2'-O-ribose methyltransferase nsp16 binding(Jiang et al., 2020)
73	Carfilzomib	D08889	-15.2	Measured activity against SARS-CoV-2 M^{pro}.(Resnick et al., 2020) Predicted M^{pro} binding(Wang, 2020)
74	IPI-549	C3984425	-15.0	…
75	Milademetan	C4292264	-14.6	Predicted M^{pro} binding(Wang, 2020)
76	Nemiralisib	C2216859	-14.4	Predicted M^{pro} binding(Bembenek, 2020)

	Name	ChEMBL (C)) or Drugbank (D) ID	ΔG_{MMPBSA} kcal/mol	SARS-Cov-2 data
77	Amrubicin	C1186894	-14.3	Predicted M^{pro} binding (Jiménez-Alberto et al., 2020)
78	Genz-10850	D04289	-13.1	Predicted nsp12 binding(Yu et al., 2020)
79	Penimepicycline	C 3833378	-12.9	Predicted to M^{pro} and spike(Durdagi et al., 2020) binding
80	Tipifarnib	C289228	-12.6	…
81	MK3207	C1910936	-12.1	Predicted M^{pro},(Olubiyi et al., 2020) PLpro,(Contreras-Puentes and Alvíz-Amador, 2020) 2'-O-ribose methyl-transferase nsp16(Jiang et al., 2020) binding
82	Naldemedine	C2105791	-12.1	Predicted M^{pro} (Subramanian, 2020) and spike RBD (Ramírez-Salinas et al., 2020) binding
83	Tariquidar	D06240	-12.0	66% reduction in M^{pro} activity at 4 μM.(Milligan et al., 2021)
84	Netupitant	C206253	-11.9	Predicted RdRp binding (Hosseini et al., 2020) and M^{pro} (Subramanian, 2020)

Table S2. Binding interactions with M^{pro} binding site for top 10 ranked drugs.

ID	Drug	Interacting Residues	H-Bond
CHEMBL7835	Bemcitinib	THR26, LEU27, HIS41, ASN142, GLY143, SER144, CYS145, HIS164, MET165, GLU166, LEU167, PRO168, VAL186, ASP187, ARG188, GLN189, THR190, ALA191, GLN192	VAL186 (O-N8) 2.70<br>ARG188(O-N8) 2.65<br>GLN192 (NE2 -N7) 3.27
CHEMBL442	Ergotamine	THR25, LEU27, HIS41, PHE140, LEU141, ASN142, GLY143, SER144, CYS145, HIS163, HIS164, MET165, GLU166, HIS172, VAL186, ASP187, ARG188, GLN189, THR190, GLN192	GLY143(N-O4) 2.68<br>HIS164 (O-O5) 3.29<br>MET165(SD-C12) 3.18
DB01601	Lopinavir	THR26, HIS41, MET49, PHE140, LEU141, ASN142, CYS145, HIS163, HIS164, MET165, GLU166, HIS172, VAL186, ASP187, ARG188, GLN189, THR190, GLN192	ASN142 (OD1-O2) 2.59

ID	Drug	Interacting Residues	H-Bond
CHEMBL4958	Mergocriptine	HIS41, CYS44, MET49, ASN142, GLY143, SER144, CYS145, HIS163, HIS164, MET165, GLU166, LEU167, PRO168, VAL186, ASP187, ARG188, GLN189, THR190, GLN192	CYS145(SG-O3) 3.22<br>THR190 (O-N5) 3.01
CHEMBL4291143	PC-786	THR25, THR26, HIS41, CYS44, MET49, TYR54, PHE140, LEU141, ASN142, GLY143, SER144, CYS145, HIS163, HIS164, MET165, GLU166, HIS172, ASP187, ARG188, GLN189	GLY143(N-O4) 2.67<br>SER144(OG-F1) 2.59<br>SER144(N-O4) 2.85<br>CYS145 (SG-F1) 3.01<br>CYS145 (N-)4) 3.06
DB14761	Remdesivir	HIS41, MET49, PHE140, LEU141, ASN142, GLY143, SER144, CYS145, HIS163, HIS164, MET165, GLU166, HIS172, VAL186, ASP187, ARG188, THR190, GLN192	PHE140(O-N5) 2.98<br>SER144(OG-N6) 3.14<br>HIS163(NE2-N6) 3.01<br>HIS164(O-O4) 2.67
DB00503	Ritonavir	THR25, THR26, LEU27, HIS41, CYS44, THR45, SER46, MET47, PHE140, LEU141, ASN142, GLY143, SER144, CYS145, HIS163, HIS164, MET165, GLU166, LEU167, PRO168, HIS172, VAL186, ARG188, GLN189, THR190, GLN192	CYS145(SG-O3) 3.03<br>HIS164(O-O3) 2.88
DB06290	Simeprevir	HIS41, CYS44, MET49, TYR54, PHE140, LEU141, ASN142, GLY143, SER144, CYS145, HIS163, HIS164, MET165, GLU166, LEU167, PRO168, THR169, GLY170, HIS172, VAL186, ARG188, GLN189, THR190, GLN192	HIS163(NE2-O4) 3.09<br>HIS164(O-N3) 3.18<br>CYS145(O-HO) 3.34
DB08934	Sofosbuvir	HIS41, MET49, TYR54, PHE140, LEU141, ASN142, GLY143, SER144, CYS145, HIS163, HIS164, MET165, GLU166, LEU167, PRO168, HIS172, VAL186, ARG188, GLN189, THR190, GLN192, ALA193	SER144(OG-O9) 3.09<br>GLU166(N-O6) 3.28

ID	Drug	Interacting Residues	H-Bond
CHEMBL4499	Montelukast	THR25, THR26, LEU27, HIS41, MET49, TYR54, PHE140, LEU141, ASN142, GLY143, SER144, CYS145, HIS163, HIS164, MET165, GLU166, LEU167, PRO168, ASP187, ARG188, GLN189, THR190, GLN192	SER144 (N-O3) 2.95<br>SER144 (OG-O3) 2.89<br>CYS145 (N-O3) 3.17

Scripts:

1)Conf.txt

```
receptor = 6Y2F.pdbqt

center_x=  9.245

center_y=  -0.788

center_z = 18.371

size_x = 50

size_y = 50

size_z = 50

num_modes = 10

exhaustiveness = 50
```

2)vina_screen.sh

```
#! /bin/bash

for f in CHEMBL*.pdbqt; do

   b=`basename $f .pdbqt`

   echo Processing ligand $b

mkdir -p $b

   vina --config conf.txt --cpu 50 --ligand $f --out $[b]/out.pdbqt --log $[b]/log.txt

done
```

3)Script1.py

```
#! /usr/bin/env python
```

```
import sys

import glob

def doit(n):

file_names = glob.glob('*/*.pdbqt')

    everything = []

    failures = []

    print 'Found', len(file_names), 'pdbqt files'

    for file_name in file_names:

        file = open(file_name)

        lines = file.readlines()

file.close()

        try:

            line = lines[1]

            result = float(line.split(':')[1].split()[0])

everything.append([result, file_name])

        except:

failures.append(file_name)

everything.sort(lambda x,y: cmp(x[0], y[0]))

    part = everything[:n]

    for p in part:

        print p[1],

    print

    if len(failures) > 0:

        print 'WARNING:', len(failures), 'pdbqt files could not be processed'
```

```
if __name__ == '__main__':

doit(int(sys.argv[1]))
```

References

Adem, S., Eyupoglu, V., Sarfraz, I., Rasul, A., and Ali, M. 2020. Identification of potent COVID-19 main protease (Mpro) inhibitors from natural polyphenols: An in silico strategy unveils a hope against CORONA. *Preprints* [Online]. Available: https://www.preprints.org/manuscript/202003.0333/v1/download.

Alamri, M.A., Tahir Ul Qamar, M., Mirza, M.U., Bhadane, R., Alqahtani, S.M., Muneer, I., Froeyen, M., and Salo-Ahen, O.M.H. (2020). Pharmacoinformatics and molecular dynamics simulation studies reveal potential covalent and FDA-approved inhibitors of SARS-CoV-2 main protease 3CL(pro). *J Biomol Struct Dyn*, 1-13.

Alexpandi, R., De Mesquita, J.F., Pandian, S.K., and Ravi, A.V. (2020). Quinolines-Based SARS-CoV-2 3CLpro and RdRp Inhibitors and Spike-RBD-ACE2 Inhibitor for Drug-Repurposing Against COVID-19: An in silico Analysis. *Front Microbiol* 11, 1796.

Allam, A.E., Assaf, H.K., Hassan, H.A., Shimizu, K., and Elshaier, Y.a.M.M. (2020). An in silico perception for newly isolated flavonoids from peach fruit as privileged avenue for a countermeasure outbreak of COVID-19. *RSC Adv,* 10, 29983-29998.

Alméciga-Díaz, C.J., Pimentel-Vera, L.N., Caro, A., Mosquera, A., Moreno, C.a.C., Rojas, J.P.M., and Díaz-Tribaldos, D.C. 2020. Virtual screening of potential inhibitors for SARS-CoV-2 main protease. *Preprints* [Online]. Available: https://www.preprints.org/manuscript/202004.0146/v1/download.

Arul, M.N., Kumar, S., Jeyakanthan, J., and Srivastav, V. 2020. Searching for target-specific and multi-targeting organics for Covid-19 in the Drugbank database with a double scoring approach. *ResearchSquare* [Online]. Available: https://assets.researchsquare.com/files/rs-36233/v1_stamped.pdf.

Athar, F., and Beg, M.A. (2020). Anti-HIV and Anti-HCV drugs are the putative inhibitors of RNA-dependent-RNA polymerase activity of NSP12 of the SARS CoV-2 (COVID-19). *Pharm Pharmacol Int J* 8, 163-172.

Beck, B.R., Shin, B., Choi, Y., Park, S., and Kang, K. (2020). Predicting commercially available antiviral drugs that may act on the novel coronavirus (SARS-CoV-2) through a drug-target interaction deep learning model. *Comput Struct Biotechnol J* 18, 784-790.

Bembenek, S. 2020. Drug Repurposing and New Therapeutic Strategies for SARS-CoV-2 Disease Using a Novel Molecular Modeling-AI Hybrid Workflow. *ChemRxiv* [Online].

Available: https://s3-eu-west-1.amazonaws.com/pstorage-chemrxiv-899408398289/23108015/bembenek_covid19_computional_models_v2.pdf.

Bolcato, G., Bissaro, M., Pavan, M., Sturlese, M., and Moro, S. (2020). Targeting the coronavirus SARS-CoV-2: computational insights into the mechanism of action of the protease inhibitors lopinavir, ritonavir and nelfinavir. *Sci Rep* 10**,** 20927.

Borgio, J.F., Alsuwat, H.S., Al Otaibi, W.M., Ibrahim, A.M., Almandil, N.B., Al Asoom, L.I., Salahuddin, M., Kamaraj, B., and Abdulazeez, S. (2020). State-of-the-art tools unveil potent drug targets amongst clinically approved drugs to inhibit helicase in SARS-CoV-2. *Arch Med Sci* 16**,** 508-518.

Bozek, A., and Winterstein, J. (2020). Montelukast's ability to fight COVID-19 infection. *J Asthma***,** 1-2.

Cao, B., Wang, Y., Wen, D., Liu, W., Wang, J., Fan, G., Ruan, L., Song, B., Cai, Y., Wei, M., Li, X., Xia, J., Chen, N., Xiang, J., Yu, T., Bai, T., Xie, X., Zhang, L., Li, C., Yuan, Y., Chen, H., Li, H., Huang, H., Tu, S., Gong, F., Liu, Y., Wei, Y., Dong, C., Zhou, F., Gu, X., Xu, J., Liu, Z., Zhang, Y., Li, H., Shang, L., Wang, K., Li, K., Zhou, X., Dong, X., Qu, Z., Lu, S., Hu, X., Ruan, S., Luo, S., Wu, J., Peng, L., Cheng, F., Pan, L., Zou, J., Jia, C., Wang, J., Liu, X., Wang, S., Wu, X., Ge, Q., He, J., Zhan, H., Qiu, F., Guo, L., Huang, C., Jaki, T., Hayden, F.G., Horby, P.W., Zhang, D., and Wang, C. (2020). A Trial of Lopinavir-Ritonavir in Adults Hospitalized with Severe Covid-19. *N Engl J Med* 382**,** 1787-1799.

Chakraborti, S., Bheemireddy, S., and Srinivasan, N. (2020). Repurposing drugs against main protease of SARS-CoV-2: mechanism based insights supported by available laboratory and clinical data, Mol Omics, 16, 474-491.

Chakraborti, S., and Srinivasan, N. (2020). Drug Repurposing Approach Targeted Against Main Protease of SARS-CoV-2 Exploiting 'Neighbourhood Behaviour'in 3D Protein Structural Space and 2D Chemical Space of Small Molecules. *ChemRxiv* [Online] chemrxiv.12057846.v2.

Chen, Y.W., Yiu, C.B., and Wong, K.Y. (2020). Prediction of the SARS-CoV-2 (2019-nCoV) 3C-like protease (3CL (pro)) structure: virtual screening reveals velpatasvir, ledipasvir, and other drug repurposing candidates. *F1000Res* 9**,** 129.

Choy, K.T., Wong, A.Y., Kaewpreedee, P., Sia, S.F., Chen, D., Hui, K.P.Y., Chu, D.K.W., Chan, M.C.W., Cheung, P.P., Huang, X., Peiris, M., and Yen, H.L. (2020). Remdesivir,

lopinavir, emetine, and homoharringtonine inhibit SARS-CoV-2 replication in vitro. *Antiviral Res* 178**,** 104786.

Chtita, S., Belhassan, A., Aouidate, A., Belaidi, S., Bouachrine, M., and Lakhlifi, T. (2021). Discovery of Potent SARS-CoV-2 Inhibitors from Approved Antiviral Drugs via Docking and Virtual Screening. *Comb Chem High Throughput Screen* 24**,** 441-454.

Contreras-Puentes, N., and Alvíz-Amador, A. (2020). Virtual Screening of Natural Metabolites and Antiviral Drugs with Potential Inhibitory Activity against 3CL-PRO and PL-PRO. *Biomed Pharmacol J* 13.

Copertino, D.C., Duarte, R.R.R., Powell, T.R., De Mulder Rougvie, M., and Nixon, D.F. (2021). Montelukast drug activity and potential against severe acute respiratory syndrome coronavirus 2 (SARS-CoV-2). *J Med Virol* 93**,** 187-189.

Costanzo, M., De Giglio, M.a.R., and Roviello, G.N. (2020). SARS-CoV-2: Recent Reports on Antiviral Therapies Based on Lopinavir/Ritonavir, Darunavir/Umifenovir, Hydroxychloroquine, Remdesivir, Favipiravir and other Drugs for the Treatment of the New Coronavirus. *Curr Med Chem* 27**,** 4536-4541.

Cozac, R., Medzhidov, N., and Yuk, S. 2020. Predicting inhibitors for SARS-CoV-2 RNA-dependent RNA polymerase using machine learning and virtual screening. *arXiv* [Online] arXiv:2006.06523.

Day, C.J., Bailly, B., Guillon, P., Dirr, L., Jen, F.E., Spillings, B.L., Mak, J., Von Itzstein, M., Haselhorst, T., and Jennings, M.P. (2021). Multidisciplinary Approaches Identify Compounds that Bind to Human ACE2 or SARS-CoV-2 Spike Protein as Candidates to Block SARS-CoV-2-ACE2 Receptor Interactions. *mBio* 12**,** e03681-03620.

De Lima Menezes, G., and Da Silva, R.A. (2020). Identification of potential drugs against SARS-CoV-2 non-structural protein 1 (nsp1). *J Biomol Struct Dyn***,** 1-11.

Dittmar, M., Lee, J.S., Whig, K., Segrist, E., Li, M., Jurado, K., Samby, K., Ramage, H., Schultz, D., and Cherry, S. 2020. Drug repurposing screens reveal FDA approved drugs active against SARS-Cov-2. *BioRxiv* [Online]. Available: https://www.biorxiv.org/content/biorxiv/early/2020/06/19/2020.06.19.161042.full.pdf.

Durdagi, S., Aksoydan, B., Dogan, B., Sahin, K., and Shahraki, A. 2020. Screening of Clinically Approved and Investigation Drugs as Potential Inhibitors of COVID-19 Main Protease: A Virtual Drug Repurposing Study. *ChemRxiv* [Online]. Available: https://s3-eu-

west-1.amazonaws.com/pstorage-chemrxiv-899408398289/22424652/COVID19_Paper_DurdagiLab_26April_2020.pdf.

Elfiky, A., Ibrahim, N., and Elshemey, W. 2020. Drug repurposing against MERS CoV and SARS-COV-2 PLpro in silico. *Research Square* [Online]. Available: https://assets.researchsquare.com/files/rs-19600/v1/manuscript.pdf.

Elfiky, A.A. (2020). Ribavirin, Remdesivir, Sofosbuvir, Galidesivir, and Tenofovir against SARS-CoV-2 RNA dependent RNA polymerase (RdRp): A molecular docking study. *Life Sci* 253**,** 117592.

Encinar, J.A., and Menendez, J.A. (2020). Potential Drugs Targeting Early Innate Immune Evasion of SARS-Coronavirus 2 via 2'-O-Methylation of Viral RNA. *Viruses* 12**,** 525.

Feng, S., Luan, X., Wang, Y., Wang, H., Zhang, Z., Wang, Y., Tian, Z., Liu, M., Xiao, Y., Zhao, Y., Zhou, R., and Zhang, S. (2020). Eltrombopag is a potential target for drug intervention in SARS-CoV-2 spike protein. *Infect Genet Evolut* 85**,** 104419.

Fintelman-Rodrigues, N., Sacramento, C.Q., Ribeiro Lima, C., Souza Da Silva, F., Ferreira, A.C., Mattos, M., De Freitas, C.S., Cardoso Soares, V., Da Silva Gomes Dias, S., Temerozo, J.R., Miranda, M.D., Matos, A.R., Bozza, F.A., Carels, N., Alves, C.R., Siqueira, M.M., Bozza, P.T., and Souza, T.M.L. (2020). Atazanavir, Alone or in Combination with Ritonavir, Inhibits SARS-CoV-2 Replication and Proinflammatory Cytokine Production. *Antimicrob Agents Chemother* 64**,** e00825-00820.

Gul, S., Ozcan, O., Asar, S., Okyar, A., Baris, I., and Kavakli, I.H. (2020). In silico identification of widely used and well-tolerated drugs as potential SARS-CoV-2 3C-like protease and viral RNA-dependent RNA polymerase inhibitors for direct use in clinical trials. *J Biomol Struct Dyn***,** 1-20.

Gurung, A.B., Ali, M.A., Lee, J., Abul Farah, M., and Al-Anazi, K.M. (2020). In silico screening of FDA approved drugs reveals ergotamine and dihydroergotamine as potential coronavirus main protease enzyme inhibitors. *Saudi J Biol Sci* 27**,** 2674-2682.

Hakmi, M., Bouricha, E., Kandoussi, I., El Harti, J., and Ibrahimi, A. (2020). Repurposing of known anti-virals as potential inhibitors for SARS-CoV-2 main protease using molecular docking analysis. *Bioinform* 16**,** 301-305.

Hosseini, M., Chen, W., and Wang, C. 2020. Computational Molecular Docking and Virtual Screening Revealed Promising SARS-CoV-2 Drugs. . *ChemRxiv* [Online] chemrxiv.12237995.v1.

Huang, D., Yu, H., Wang, T., Yang, H., Yao, R., and Liang, Z. (2021). Efficacy and safety of umifenovir for coronavirus disease 2019 (COVID-19): A systematic review and meta-analysis. *J Med Virol* 93**,** 481-490.

Huang, H., Guan, L., Yang, Y., Le Grange, J.M., Tang, G., Xu, Y., Yuan, J., Lin, C., Xue, M., Zhang, X., Chen, R., Zhou, L., and Huang, W. 2020. Chloroquine, arbidol (umifenovir) or lopinavir/ritonavir as the antiviral monotherapy for COVID-19 patients: a retrospective cohort study. *ResearchSquare* [Online]. Available: https://assets.researchsquare.com/files/rs-24667/v1/manuscript.pdf.

Jacome, R., Campillo-Balderas, J.A., Ponce De Leon, S., Becerra, A., and Lazcano, A. (2020). Sofosbuvir as a potential alternative to treat the SARS-CoV-2 epidemic. *Sci Rep* 10**,** 9294.

Jan, J.T., Cheng, T.R., Juang, Y.P., Ma, H.H., Wu, Y.T., Yang, W.B., Cheng, C.W., Chen, X., Chou, T.H., Shie, J.J., Cheng, W.C., Chein, R.J., Mao, S.S., Liang, P.H., Ma, C., Hung, S.C., and Wong, C.H. (2021). Identification of existing pharmaceuticals and herbal medicines as inhibitors of SARS-CoV-2 infection. *Proc Natl Acad Sci U S A* 118**,** e2021579118.

Jiang, Y., Liu, L., Manning, M., Bonahoom, M., Lotvola, A., and Yang, Z.-Q. 2020. Repurposing Therapeutics to Identify Novel Inhibitors Targeting 2'-O-Ribose Methyltransferase Nsp16 of SARS-CoV-2. *ChemRxiv* [Online] chemrxiv.12252965.v1.

Jiménez-Alberto, A., Ribas-Aparicio, R.M., Aparicio-Ozores, G., and Castelán-Vega, J.A. (2020). Virtual screening of approved drugs as potential SARS-CoV-2 main protease inhibitors. *Computational Biology and Chemistry***,** 107325.

Kandwal, S., and Fayne, D. 2020. Repurposing drugs for treatment of SARS-CoV-2 infection: Computational design insights into mechanisms of action. *ResearchSquare* [Online]. Available: https://assets.researchsquare.com/files/rs-54535/v1/bbc9054a-231b-4ab6-8485-2f08990e7f20.pdf.

Khater, S., Dasgupta, N., and Das, G. 2020. Combining SARS-cov-2 Proofreading Exonuclease and RNA-dependent RNA Polymerase Inhibitors as a Strategy to Combat COVID-19: A High-throughput in Silico Screen. *OSF Preprints* [Online] osf.io/7x5ek..

Ko, M., Jeon, S., Ryu, W.S., and Kim, S. (2021). Comparative analysis of antiviral efficacy of FDA-approved drugs against SARS-CoV-2 in human lung cells. *J Med Virol* 93**,** 1403-1408.

Kouznetsova, V., Huang, D., and Tsigelny, I.F. (2021). Potential COVID-19 Mpro Inhibitors: Repurposing FDAapproved Drugs. *Phys Biol.* 18**,** 025001.

Kumar, S., Singh, B., Kumari, P., Kumar, P.V., Agnihotri, G., Khan, S., Kant Beuria, T., Syed, G.H., and Dixit, A. (2021). Identification of multipotent drugs for COVID-19 therapeutics with the evaluation of their SARS-CoV2 inhibitory activity. *Comput Struct Biotechnol J* 19**,** 1998-2017.

Li, Y., Zhang, Y., Han, Y., Zhang, T., and Du, R. 2020. Prioritization of Potential Drugs Targeting the SARS-CoV-2 Main Protease. *ChemRxiv* [Online] chemrxiv.12629858.v1.

Lo, H.S., Hui, K.P., Lai, H.-M., Khan, K.S., Kaur, S., Li, Z., Chan, A.K., Cheung, H.H.-Y., Ng, K.C., and Ho, J.C.W. 2020. Simeprevir suppresses SARS-CoV-2 replication and synergizes with remdesivir. *bioRxiv* [Online]. Available: https://www.biorxiv.org/content/biorxiv/early/2020/09/03/2020.05.26.116020.full.pdf.

Massignan, T., Boldrini, A., Terruzzi, L., Spagnolli, G., Astolfi, A., Bonaldo, V., Pischedda, F., Pizzato, M., Lolli, G., Barreca, M.L., Biasini, E., Faccioli, P., and Pieri, L. 2021. Antimalarial Artefenomel Inhibits Human SARS-CoV-2 Replication in Cells while Suppressing the Receptor ACE2. *arXiv* [Online] 2004.13493v4.

Milligan, J.C., Zeisner, T.U., Papageorgiou, G., Joshi, D., Soudy, C., Ulferts, R., Wu, M., Lim, C.T., Tan, K.W., Weissmann, F., Canal, B., Fujisawa, R., Deegan, T., Nagaraj, H., Bineva-Todd, G., Basier, C., Curran, J.F., Howell, M., Beale, R., Labib, K., O'reilly, N., and Diffley, J.F.X. (2021). Identifying SARS-CoV-2 antiviral compounds by screening for small molecule inhibitors of Nsp5 main protease. *Biochem J* 478**,** 2499-2515.

Muralidharan, N., Sakthivel, R., Velmurugan, D., and Gromiha, M.M. (2021). Computational studies of drug repurposing and synergism of lopinavir, oseltamivir and ritonavir binding with SARS-CoV-2 protease against COVID-19. *J Biomol Struct Dyn* 39**,** 2673-2678.

Naveen, S.M., and Reddy, M.S. 2020. Target SARS-CoV-2: Computation of Binding energies with drugs of Dexamethasone/Umifenovir by Molecular Dynamics using OPLS-AA force field. *Research Square* [Online] rs-40785/v1.

Olender, S.A., Perez, K.K., Go, A.S., Balani, B., Price-Haywood, E.G., Shah, N.S., Wang, S., Walunas, T.L., Swaminathan, S., Slim, J., Chin, B., De Wit, S., Ali, S.M., Soriano Viladomiu, A., Robinson, P., Gottlieb, R.L., Tsang, T.Y.O., Lee, I.H., Haubrich, R.H., Chokkalingam, A.P., Lin, L., Zhong, L., Bekele, B.N., Mera-Giler, R., Gallant, J., Smith, L.E., Osinusi, A.O., Brainard, D.M., Hu, H., Phulpin, C., Edgar, H., Diaz-Cuervo, H., and Bernardino, J.I. (2020). Remdesivir for Severe COVID-19 versus a Cohort Receiving Standard of Care. *Clin Infect Dis***,** ciaa1041.

Olubiyi, O.O., Olagunju, M., Keutmann, M., Loschwitz, J., and Strodel, B. (2020). High Throughput Virtual Screening to Discover Inhibitors of the Main Protease of the Coronavirus SARS-CoV-2. *Molecules* 25.

Omotuyi, O., Nash, O., Ajiboye, B., Metibemu, D., Oyinloye, B., and Ojo, A. 2020. The disruption of SARS-CoV-2 RBD/ACE-2 complex by Ubrogepant Is mediated by interface hydration. *Preprints* [Online] preprints202003.0466.v1)..

Panda, P.K., Arul, M.N., Patel, P., Verma, S.K., Luo, W., Rubahn, H.G., Mishra, Y.K., Suar, M., and Ahuja, R. (2020). Structure-based drug designing and immunoinformatics approach for SARS-CoV-2. *Sci Adv* 6**,** eabb8097.

Peterson, L. (2020). In Silico Molecular Dynamics Docking of Drugs to the Inhibitory Active Site of SARS-CoV-2 Protease and Their Predicted Toxicology and ADME. *ChemRxiv, chemrxiv.12155523.v1*.

Pirzada, R.H., Haseeb, M., Batool, M., Kim, M., and Choi, S. (2021). Remdesivir and Ledipasvir among the FDA-Approved Antiviral Drugs Have Potential to Inhibit SARS-CoV-2 Replication. *Cells* 10**,** 1052.

Qiao, Z., Zhang, H., Ji, H.F., and Chen, Q. (2020). Computational View toward the Inhibition of SARS-CoV-2 Spike Glycoprotein and the 3CL Protease. *Computation* 8**,** 53.

Ramírez-Salinas, G.L., MartíNez-Archundia, M., Correa-Basurto, J., and García-Machorro , J. 2020. Repositioning of ligands that target spike glycoprotein as potential drugs against SARS-CoV-2. *ResearchSquare* [Online] s.3.rs-52025/v1.

Rehman, M.T., Alajmi, M.F., and Hussain, A. 2020. Natural Compounds as Inhibitors of SARS-CoV-2 Main Protease (3CLpro): A Molecular Docking and Simulation Approach to Combat COVID-19. *ChemRxiv* [Online] chemrxiv.12362333.v2..

Reig, N., and Shin, D.-H. (2020). 560. Repurposing Eravacycline for the Treatment of SARS-CoV-2 Infections. *Open Forum Infectious Diseases* 7**,** S345-S345.

Resnick, S.J., Iketani, S., Hong, S.J., Zask, A., Liu, H., Kim, S., Melore, S., Nair, M.S., Huang, Y., Tay, N.E.S., Rovis, T., Yang, H.W., Stockwell, B.R., Ho, D.D., and Chavez, A. (2020). A simplified cell-based assay to identify coronavirus 3CL protease inhibitors. *bioRxiv***,** 2020.2008.2029.272864.

Romeo, A., Iacovelli, F., and Falconi, M. (2020). Targeting the SARS-CoV-2 spike glycoprotein prefusion conformation: virtual screening and molecular dynamics simulations applied to the identification of potential fusion inhibitors. *Virus Res* 286**,** 198068.

Ruan, Z., Liu, C., Guo, Y., He, Z., Huang, X., Jia, X., and Yang, T. (2021). SARS-CoV-2 and SARS-CoV: Virtual screening of potential inhibitors targeting RNA-dependent RNA polymerase activity (NSP12). *J Med Virol* 93**,** 389-400.

Sacramento, C., Fintelman-Rodrigues, N., Temerozo, J.R., Da Silva Gomes Dias, S., Ferreira, A.C., Mattos, M., Pão, C.R.R., De Freitas, C.S., Soares, V.C., Bozza, F.A., Bou-Habib, D.C., Bozza, P.T., and T.M.L., S. 2020. The in vitro antiviral activity of the anti-hepatitis C virus (HCV) drugs daclatasvir and sofosbuvir against SARS-CoV-2. *bioRxiv* [Online] 2020.06.15.153411.

Sekhar, T. 2020. Virtual Screening based prediction of potential drugs for COVID-19. *Preprints* [Online] preprints202002.0418.v2.

Senathilake, K., Samarakoon, S., and Tennekoon, K. 2020. Virtual Screening of Inhibitors Against Spike Glycoprotein of SARS-CoV-2: A Drug Repurposing Approach. *Preprints* [Online] preprints202003.0042.v2.

Sharma, K., Morla, S., Goyal, A., and Kumar, S. (2020). Computational guided drug repurposing for targeting 2'-O-ribose methyltransferase of SARS-CoV-2. *Life Sci* 259**,** 118169.

Sobeh, M., Mrid, R.B., and Yasri, A. Virtual Screening of Heterocyclic Molecules to Identify Potential SARS-COV2 virus Mpro Protease Inhibitors for Further Medicinal Chemistry design *Research Square* [Online] rs.3.rs-37557/v1.

Subramanian, S. 2020. Some FDA Approved Drugs Exhibit Binding Affinity as High as -16.0 Kcal/mol Against COVID-19 Main Protease (mpro): A Molecular Docking Study. *IndiaRxiv* [Online] osf.io/t7jsd..

Vafaei, S., Razmi, M., Mansoori, M., Asadi-Lari, M., and Madjd, Z. (2020). Spotlight of Remdesivir in Comparison with Ribavirin, Favipiravir, Oseltamivir and Umifenovir in Coronavirus Disease 2019 (COVID-19) Pandemic. *SSRN Electronic Journal, DOI: 10.2139/ssrn.3569866*.

Verdugo-Paiva, F., Izcovich, A., Ragusa, M., and Rada, G. (2020). Lopinavir-ritonavir for COVID-19: A living systematic review. *Medwave* 20**,** e7967.

Wang, J. (2020). Fast Identification of Possible Drug Treatment of Coronavirus Disease-19 (COVID-19) through Computational Drug Repurposing Study. *J Chem Inf Model* 60**,** 3277-3286.

Wang, M., Cao, R., Zhang, L., Yang, X., Liu, J., Xu, M., Shi, Z., Hu, Z., Zhong, W., and Xiao, G. (2020a). Remdesivir and chloroquine effectively inhibit the recently emerged novel coronavirus (2019-nCoV) in vitro. *Cell Res* 30**,** 269-271.

Wang, Y., Zhang, D., Du, G., Du, R., Zhao, J., Jin, Y., Fu, S., Gao, L., Cheng, Z., Lu, Q., Hu, Y., Luo, G., Wang, K., Lu, Y., Li, H., Wang, S., Ruan, S., Yang, C., Mei, C., Wang, Y., Ding, D., Wu, F., Tang, X., Ye, X., Ye, Y., Liu, B., Yang, J., Yin, W., Wang, A., Fan, G., Zhou, F., Liu, Z., Gu, X., Xu, J., Shang, L., Zhang, Y., Cao, L., Guo, T., Wan, Y., Qin, H., Jiang, Y., Jaki, T., Hayden, F.G., Horby, P.W., Cao, B., and Wang, C. (2020b). Remdesivir in adults with severe COVID-19: a randomised, double-blind, placebo-controlled, multicentre trial. *Lancet* 395**,** 1569-1578.

Wilkinson, T., Dixon, R., Page, C., Carroll, M., Griffiths, G., Ho, L.P., De Soyza, A., Felton, T., Lewis, K.E., Phekoo, K., Chalmers, J.D., Gordon, A., Mcgarvey, L., Doherty, J., Read, R.C., Shankar-Hari, M., Martinez-Alier, N., O'kelly, M., Duncan, G., Walles, R., Sykes, J., Summers, C., Singh, D., and Collaborators, A. (2020). ACCORD: A Multicentre, Seamless, Phase 2 Adaptive Randomisation Platform Study to Assess the Efficacy and Safety of Multiple Candidate Agents for the Treatment of COVID-19 in Hospitalised Patients: A structured summary of a study protocol for a randomised controlled trial. *Trials* 21**,** 691.

Xiao, X., Wang, C., Chang, Wang, Y., Dong, X., Jiao, T., Zhao, Z., Ren, L., Dela Cruz, C.S., Sharma, L., Lei, X., and Wang, J. (2020). Identification of Potent and Safe Antiviral Therapeutic Candidates Against SARS-CoV-2. *Front Immunol* 11**,** 586572.

Xie, X., Muruato, A.E., Zhang, X., Lokugamage, K.G., Fontes-Garfias, C.R., Zou, J., Liu, J., Ren, P., Balakrishnan, M., Cihlar, T., Tseng, C.-T.K., Makino, S., Menachery, V.D., Bilello,

J.P., and Shi, P.-Y. 2020. A nanoluciferase SARS-CoV-2 for rapid neutralization testing and screening of anti-infective drugs for COVID-19. *bioRxiv* [Online] 2020.06.22.165712.

Yamamoto, N., Matsuyama, S., Hoshino, T., and Yamamoto, N. 2020. Nelfinavir inhibits replication of severe acute respiratory syndrome coronavirus 2 in vitro. *bioRxiv* [Online] 2020.04.06.026476.

Yu, J., Shao, S., Liu, B., Wang, Z., Jiang, Y.-Z., Li, Y., Chen, F., and Liu, B. 2020. Emergency Antiviral Drug Discovery During a Pandemic-a Case Study on the Application of Natural Compounds to Treat COVID-19. *ChemRxiv* [Online] chemrxiv.12307592.v1.

Yu, S., Sun, G., Sui, Y., Li, H., Zhang, N., Bi, Y., Gao, G., Jiang, L., Xu, P., Yuan, C., Yang, Y., and Huang, M. (2021). Potent Inhibition of Severe Acute Respiratory Syndrome Coronavirus 2 (SARS-CoV-2) by photosensitizers. *Research Square, DOI: 10.21203/rs.3.rs-152985/v1.*

Zeng, J., Weissmann, F., Bertolin, A.P., Posse, V., Canal, B., Ulferts, R., Wu, M., Harvey, R., Hussain, S., Milligan, J.C., Roustan, C., Borg, A., Mccoy, L., Drury, L.S., Kjaer, S., Mccauley, J., Howell, M., Beale, R., and Diffley, J.F.X. (2021). Identifying SARS-CoV-2 antiviral compounds by screening for small molecule inhibitors of nsp13 helicase. *Biochem J* 478**,** 2405-2423.
